# Interventions to improve the quality of bystander cardiopulmonary resuscitation: A systematic review

**DOI:** 10.1371/journal.pone.0211792

**Published:** 2019-02-13

**Authors:** Kuan-Yu Chen, Ying-Chih Ko, Ming-Ju Hsieh, Wen-Chu Chiang, Matthew Huei-Ming Ma

**Affiliations:** 1 College of Medicine, National Taiwan University, Taipei, Taiwan; 2 Department of Emergency Medicine, National Taiwan University Hospital, Taipei, Taiwan; 3 Department of Emergency Medicine, National Taiwan University Hospital Yun-Lin Branch, Yun-Lin County, Taiwan; St. Michael’s Hospital, CANADA

## Abstract

**Background:**

Performing high-quality bystander cardiopulmonary resuscitation (CPR) improves the clinical outcomes of victims with sudden cardiac arrest. Thus far, no systematic review has been performed to identify interventions associated with improved bystander CPR quality.

**Methods:**

We searched Ovid MEDLINE, Ovid EMBASE, EBSCO CINAHL, Ovid PsycInfo, Thomson Reuters SCI-EXPANDED, and the Cochrane Central Register of Controlled Trials to retrieve studies published from 1 January 1966 to 5 October 2018 associated with interventions that could improve the quality of bystander CPR. Data regarding participant characteristics, interventions, and design and outcomes of included studies were extracted.

**Results:**

Of the initially identified 2,703 studies, 42 were included. Of these, 32 were randomized controlled trials. Participants included adults, high school students, and university students with non-medical professional majors. Interventions improving bystander CPR quality included telephone dispatcher-assisted CPR (DA-CPR) with simplified or more concrete instructions, compression-only CPR, and other on-scene interventions, such as four-hand CPR for elderly rescuers, kneel on opposite sides for two-person CPR, and CPR with heels for a tired rescuer. Devices providing real-time feedback and mobile devices containing CPR applications or software were also found to be beneficial in improving the quality of bystander CPR. However, using mobile devices for improving CPR quality or for assisting DA-CPR might cause rescuers to delay starting CPR.

**Conclusions:**

To further improve the clinical outcomes of victims with cardiac arrest, these effective interventions may be included in the guidelines for bystander CPR.

## Introduction

Sudden cardiac arrest (SCA) poses a significant threat to our community in the industrialized world and is responsible for 420,000 and 275,000 deaths per year in the US and Europe, respectively [1,2]. It has been proved that bystander cardiopulmonary resuscitation (CPR) improves the survival rate of victims with SCA [3]. Therefore, numerous strategies have been implemented to increase the rate of bystander CPR. For example, many CPR training courses have been held to enable more laypersons to perform CPR because wider dissemination of CPR training might ultimately increase the rate of bystander CPR. Additionally, dispatcher-assisted CPR programs had also been shown to increase bystander CPR rates and the clinical outcomes of victims with SCA [4,5], and were recommended to be integrated into the system of care for prehospital cardiac arrest [6–8]. In addition to improving the rate of performing bystander CPR, the quality of CPR is also vital for the outcome of victims with SCA. High-quality CPR is associated with survival to emergency department arrival [9], survival to hospital admission [10], survival to hospital discharge [11,12], and favourable functional outcome [12]. To further improve the outcomes of victims with SCA, it is necessary to explore which interventions can improve the quality of bystander CPR. Therefore, the aim of our study was to perform a systematic review to identify interventions that could improve the quality of bystander CPR.

## Materials and methods

### Population, intervention, comparator and outcome question

We conducted a systematic review by using a predetermined protocol and reported it according to the PRISMA (Preferred Reporting Items for Systematic Reviews and Meta-analyses) guidelines [13]. The Population, Intervention, Comparator, Outcome (PICO) question of our study was: for laypersons who perform bystander CPR either in a real resuscitation situation or in a simulation setting (P), what interventions or methods except education (I), compared with no such interventions or methods (C), improved the quality of CPR (O)?

### Eligibility criteria

To answer our PICO question, the inclusion criteria for our systematic review were as follows: (1) studies addressing the question “can the intervention improve the quality of bystander CPR?”; (2) studies that were randomized controlled trials (RCTs), quasi-experimental studies, before-and-after interventional studies, crossover studies, and prospective/retrospective observational studies; (3) studies that were published after 1966; (4) original articles, articles in press or short communications; (5) studies in which the participants were laypersons; (6) studies in which the setting was a real resuscitation situation or a simulation setting; (7) studies in which the outcome measures had at least one CPR quality parameter (the CPR quality parameters considered in this study were described below: chest compression depth, chest compression rate, number of chest compression, chest recoil, interruption time during CPR, ventilation volume, time to first compression/ventilation, and correct hand positioning); (8) studies focusing on the correlation between the intervention and bystander CPR quality; and (9) studies written in English.

The exclusion criteria were as follows: (1) conference abstracts or articles, reviews, editorials, erratum, letters, case studies, and case reports; (2) non-human studies; (3) studies in which the participants included both laypersons and non-laypersons; (4) studies that provided survival outcomes instead of CPR quality data, which were required for this review.

The layperson in our study was defined as an individual who did not work at a hospital. Therefore, we did not include studies whose participants were doctors, dentists, nurses, emergency medical technicians, and pharmacists. We also excluded studies in which the participants were students who majored in medical, nursing or associated medical professionals. In addition, studies in which participants whose duty is to save lives, such as lifeguards and first responders in a public place, were also excluded.

### Information sources

We searched Ovid MEDLINE, Ovid EMBASE, EBSCO CINAHL, Ovid PsycInfo, Thomson Reuters SCI-EXPANDED, and the Cochrane Central Register of Controlled Trials (CENTRAL) to acquire studies, which could answer our PICO question. The time range was set from 1966 because 1966 was the year when the American Heart Association (AHA) published the first guidelines for CPR [14]. The last time we searched was 5 October, 2018. We also checked the reference lists of studies included for additional relevant articles.

#### Search

Our search strategy consisted of three key concepts, including cardiac arrest, bystander/layperson, and the quality of CPR. Analogous terms for each were also used. A full search strategy is provided in S1 Table in Supplementary materials.

### Study selection

Two reviewers (KYC and MJH) performed the database searching and screened out papers that were potentially relevant by reviewing titles and abstracts independently.

The article would receive full-text assessment if one of the reviewers determined that it was needed. If two reviewers had different opinions during the process of determining which article ought to be included in the final analysis, they reached an agreement after full discussion.

### Data collection process and data items

After identifying the final papers included, we collected the data, using a standard data extraction form specifically adapted for this review. Extracted data included author(s), publication year, nation, identity of the participants, date of enrolment, study design, study group, outcome, evaluation methods, and funding sources.

The outcomes among studies varied considerably. The AHA 2015 guidelines recommended high-quality CPR required adequate chest compression depth (50–60 mm), adequate chest compression rate (100–120/min), full chest wall recoil, minimal pauses in chest compressions, correct hand position during compressions, and avoidance of hyperventilation [6]. In order to focus on the essential parameters of CPR quality which the guidelines recommended, we only extracted data on compression depth, chest compression rate, number of chest compressions, chest recoil, time of interruption, ventilation, time to first compression/ventilation, and correct hand positioning.

### Risk of bias for individual studies

We used two tools to assess studies included. We used the “Cochrane Collaboration’s tool for assessing risk of bias” to assess the quality of studies for randomized controlled studies [15] and Newcastle-Ottawa Scale (NOS) for non-randomized controlled studies [16].

## Results

### Study selection

**Fig 1:**
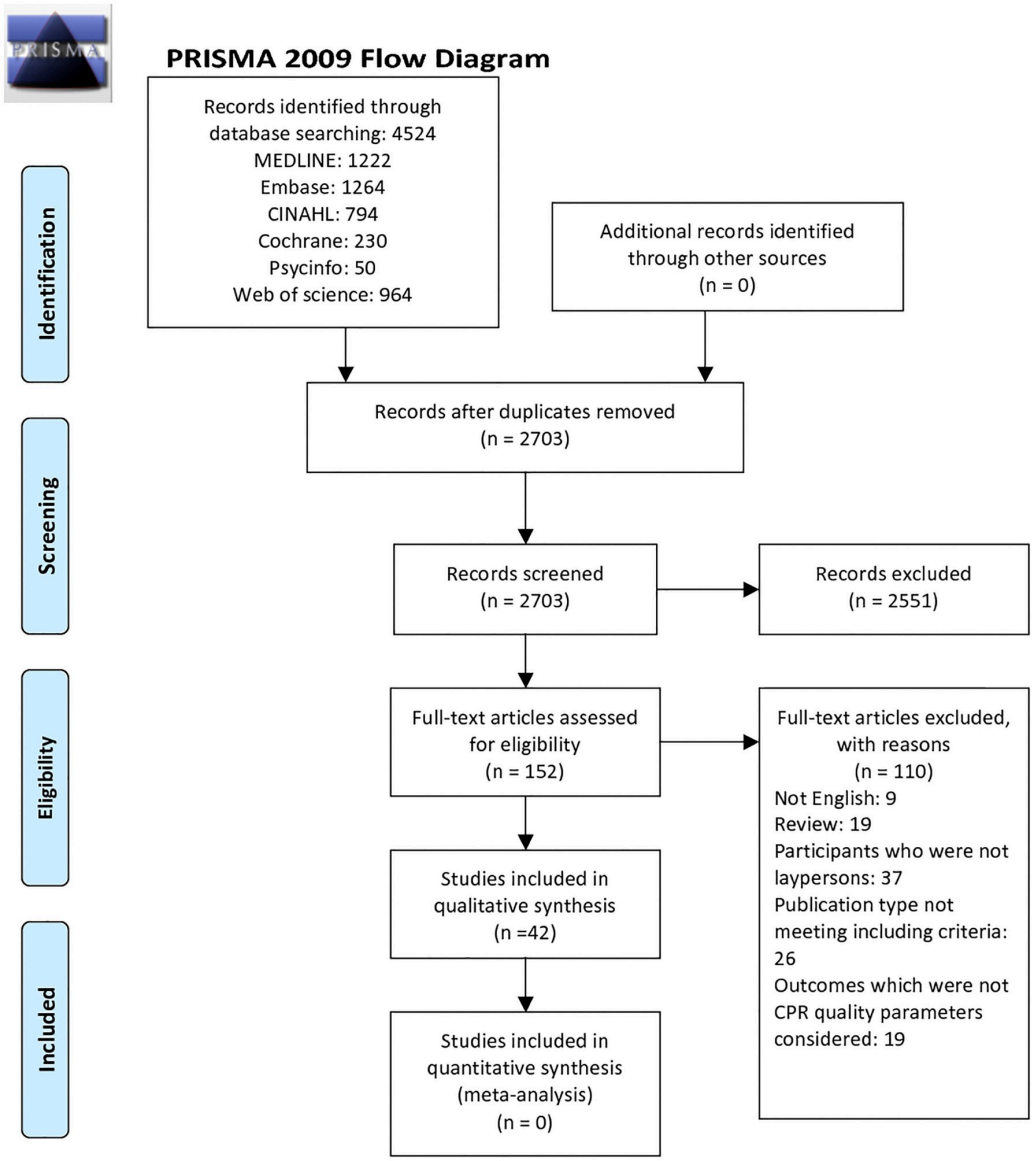
Flow diagram of included studies. *From*: Moher D, Liberati A, Tetzlaff J, Altman DG, The PRISMA Group (2009). *P*referred *R*eporting *I*terns for *S*ystematic Reviews and *M*eta-*A*nalyses: The PRISMA Statement. PLoS Med 6(6): e1000097. doi:10.1371/journal.pmed1000097. **For more information, visit** http://www.prisma-statement.org.

After the initial database searching, a total of 4,524 records were retrieved. After removing 1,821 duplicates, 2,703 articles were screened by reviewing the titles and abstracts. We then found 152 potentially relevant articles. After we reviewed the full text of these articles and reached an agreement between the two reviewers, 42 articles were finally included in our study [17–58]. The reasons for exclusion included papers that were not in English (9), review articles (19), those included participants who were not laypersons (37), publication types that do not meet the inclusion criteria (26), and those articles with outcomes which were not CPR quality parameters considered in this study (19). The flow diagram of the articles included is shown in Fig 1. The different study designs, interventions and types of data presentations in included articles precluded further meta-analysis. For example, the authors tried to evaluate whether changing some instructional content of telephone dispatcher-assisted CPR would improve bystander CPR quality in some included studies, but those changes were different among studies [22,26,27,33–35,37,40,41,52]. In other studies, the authors tried to compare the effect of compression-only CPR with that of conventional CPR on bystander CPR quality, but they presented different types of outcome data [18,25,32]. Therefore, a narrative review was performed instead.

### Characteristics and risk of bias of included studies

The characteristics and outcomes of studies included are shown in Table 1. More detailed information about characteristics and outcomes of included studies are also shown in S2 and S3 Tables in Supplementary materials. Among 42 studies included, 32 were RCTs, 6 studies [18,36,45,54,55,57] were randomized crossover controlled trials and 4 studies [24,48,49,53] were non-randomized studies. There were 13 studies conducted in North America [18,22–24,34,35,37,40,44,49,52,53,58], 19 in Europe [17,25–27,29–31,33,38,39,41–43,45,46,48,51,55,57], and 10 in the Asia-Pacific region [19–21,28,32,36,47,50,54,56]. The years of publication ranged from 1989 to 2018. Twelve studies only included participants who did not receive any CPR training previously [17,21,23,25,36,39,43,44,45,50,51,56]. The methods to evaluate CPR quality included on-scene evaluation by evaluators, observation of the video by evaluators after studies were completed, or the data acquired from the manikins with software, which could record the CPR performance of the participants.

**Table 1:** The characteristics and outcomes of included studies.

First author (year published) nation	Study design	Study Group	Evaluation methods	Outcomes evaluated by the reviewers	Outcomes from the records of manikins or computers
Kellermann A et al (1989)USA [49]	Non-RCT	(A) volunteers without prior CPR training with telephone instruction (n = 65) (B) previously trained volunteers with telephone instruction (n = 43) (C) previously trained volunteers without telephone instruction (n = 43)	Evaluated by instructors and recording manikins	Group A vs Group B vs Group C Depth: A>C, B>C (*p*<0.001) Compression rate: A>C, B>C (*p*<0.02) Generally, A = B>C Mean time to first ventilations (min): A:B:C = 2:38 vs 2:29 vs 1:03 (*p*<0.001) Mean time to first compression (mins): A:B:C = 4.07 vs 3:53 vs 1:15 (*p*<0.001) Correct hand position: A>C, B>C (*p*<0.05)	Group A vs Group B vs Group C Mean depth (mm): 18.1 vs 18.4 vs 12.4 (*p* = 0.002) Mean compression rate (n/min): 58 vs 62 vs 77 (*p*<0.001) Percentage of adequate compressions: 26.9 vs 34.4 vs 12.6 (*p* = 0.005) Mean ventilation volume (L): 0.70 vs 1.00 vs 0.98 (*p* = 0.14) Percentage of adequate ventilations: 36.0 vs 52.7 vs 58.3 (*p* = 0.02)
Woollard M et al (2003) UK [25]	RCT	(A) compression-only telephone CPR group (n = 29) (B) standard telephone CPR group (n = 30)	Observation of the video recording and measurements from a CPR training manikin with software	Group A vs Group B Ventilation: Airway opening: 64% vs 50% (*p* = 0.293) Check for airway obstruction: 84% vs 80% (*p* = 0.530) Breathing check: 76% vs 47% (*p* = 0.019) Median time to first compression (sec): 184 vs 245 (*p*<0.001) Median number of chest compressions delivered during test: 461 vs 186 (*p*<0.001)	Group A vs Group B Proportion of subjects compressing at correct depth (40–50 mm): 17% vs 7% (*p* = 0.153) Proportion of subjects compressing at correct rate (90–110 per min): 21% vs 13% (*p* = 0.343) Deliver correct breath volume: not applicable vs 17% Correct hand position: 14% vs 38% (*p* = 0.042) No evidence of exhaustion in both groups
Williams JG et al (2006) USA [44]	RCT	(A) subjects receiving traditional telephone CPR (n = 25) (B) subjects receiving compressions-only telephone CPR (n = 25)	Stopwatch used to measure time to first compression and recording strips from manikin	Group A vs Group B Percentage of time paused in 1^st^ 3 min of CPR: 36 vs 13 (*p*<0.001) Time to first compression (s): 117 vs 72 (*p*<0.001)	Group A vs Group B Total number of correct compressions: 10 vs 13 (non-significant) Compression rate (/min): 60 vs 58 (non-significant) No evidence of exhaustion in both groups
Dias JA et al (2007) USA [34]	RCT	(A) subjects given standard compression only-CPR (CC-CPR) protocol (n = 59) (B) subjects given simplified CC-CPR protocol (n = 58)	Skillreportermanikin	Group A vs Group B Time to start of compressions (sec): 78.6 vs 60.9 (*p*<0.001)	Group A vs Group B Percentage of chest compressions to the correct depth: 3% vs 31% (*p*<0.01) Mean depth of compressions (mm): 29.7 vs 35.6 (*p*<0.01) Compression rate(/min): 94 vs 104 (*p* = 0.13) Proportion with full chest recoil: 1 (0.99–1) vs 1 (0.98–1) (*p* = 0.09) Total hands-off chest time (sec): 95 vs 69 (*p*<0.001) Percentage of correct hand position: 84% vs 35% (*p*<0.01)
Brown TB et al (2008) USA [22]	RCT	(A) subjects without receiving “put the phone down” instructions (n = 108) (B) subjects receiving “put the phone down” instructions (n = 107)	Stopwatch and data from the manikin		Group A vs Group BMean compression depth (mm): 33.6 vs 32.8 (*p* = 0.60) Percentage of compressions done to correct depth: 7% vs 12% (*p* = 0.67) Mean compression rate (/min): 97.7 vs 98.8 (*p* = 0.82) Percentage of compressions done with full chest recoil: 1 (0.99–1) vs 1(1–1) (*p* = 0.05) Total hands-off chest time (sec): 71.0 vs 73.0 (*p* = 0.48) Time to start of compressions (sec): 65.0 vs 65.0 (*p* = 0.96) Percentage of compressions with correct hand position: 0.87 vs 0.90 (*p* = 0.86)
Mirza M et al (2008) USA [37]	RCT	(A) subjects with the instruction “push down firmly 2 inches” (n = 168) (B) subjects with the instruction “push as hard as you can” (n = 164)	Stopwatch and data from the manikin		Group A vs Group B Mean compression depth (mm): 29.7 vs 36.4 (*p*<0.001) Proportion of compressions done without error: 0% vs 5% (*p* = 0.003) Proportion of compressions done to correct depth: 1% vs 32% (*p*<0.001) Mean Compression rate (/min): 97.5 vs 99.7 (*p* = 0.56) Proportion of compressions done with full chest recoil: 100% vs 100% (*p* = 0.14)
Nikandish R et al (2008) Iran [36]	RCT with crossover study	(A) dominant hand group (n = 59) (B) non-dominant hand group (n = 59)	Recording manikin		Group A vs Group B Total number of compressions with inadequate depth (mean): 197 vs 196 (*p* = 0.9) Total number of compressions with incorrect hand placement (mean): 45 vs 64 (*p* = 0.1)
Yang CW et al (2008) Taiwan [28]	RCT	(A) voice group: only voice CPR instruction via a cell phone (n = 53) (B) video group: interactive voice and video instruction via a cell phone (n = 43)	Video evaluated by 2 emergency physicians and data from the computer (manikin).	Group A vs Group B Opening the airway properly: 58.5% vs 95.3% (*p*<0.01) Visible chest rise: 28.3% vs 65.1% (*p*<0.01) Open airway while giving ventilation: 60.4% vs 88.4% (*p*<0.01)	Group A vs Group B Mean volume of ventilation (ml): 322.0 vs 520.5 (*p*<0.01) Time to first rescue breath (sec): 102.0 vs 139.0 (*p*<0.01)
Bolle SR et al (2009) Norway [46]	RCT	(A) audio group: non-loudspeaker audio-call instruction (n = 26) (B) video group: loudspeaker video-assisted instruction (n = 29) * The study paired 3 into one group.	Video and data from Skillreporter manikin		Group A vs Group B Mean depth (mm): 38 vs 37 (*p* = 0.83) Done to correct depth (38–51 mm): 31% vs 35% (*p* = 0.53) Mean compression rate: (/min): 110 vs 114 (*p* = 0.75) Done with full chest recoil: 100% vs 100% (*p* = 0.83) Total hands-off-chest time (s): 331 vs 303 (*p* = 0.05) Mean ventilation volume (ml): 1356 vs 1163 (*p* = 0.74) Total number of ventilations: 24 vs 28 (*p* = 0.50) Percentage of correct volume (500–800 ml): 6% vs 11% (*p* = 0.30) Time to start of compressions (sec): 102 vs 104 (*p* = 0.29) Correct hand position: 50% vs 45% (*p* = 0.52)
Yang CW et al (2009) Taiwan [20]	RCT	(A) voice group: only voice CPR instruction via a cell phone (n = 53) (B) video group: interactive voice and video instruction via a cell phone (n = 43)	Video and data from Skillreporter manikin		Group A vs Group B Median compression depth (mm): 25.0 vs 36.0 (*p*<0.01) Median percentage of chest compressions with correct depth: 0% vs 20.0% (*p*<0.01) Mean Compression rate (/min): 63.0 vs 95.5 (*p*<0.01) Chest compressions with sufficient rate: 30.2% vs 46.5% (non-significant) Hands-off time (s): 0 vs 5.0 (*p*<0.01) Time to start of compressions (sec): 116.0 vs 145.0 (*p*<0.01) Median percentage of chest compressions with correct hand positioning: 95.6% vs 84.0% (non-significant) Median total instruction time (s): 121.0 vs 150.0 (*p*<0.01)
Merchant RM et al (2010) USA [23]	RCT	(A) CPR trained, receiving a telephone aid (n = 42) (B) CPR trained, not receiving a telephone aid (n = 40) (C) no CPR training history, receiving a cell telephone aid (n = 38) (D) no CPR training history, not receiving a cell telephone aid (n = 40)	Videotape evaluated by two authors and data from Skillreporter manikin	Group A + C vs Group B+D Total pauses (s): 74 vs 89 Time to start of compressions (sec): 48 vs 18	Group A + C vs Group B+D Mean depth (mm): 41 vs 31 Sufficient depth (38-51mm): 49% vs 31% Mean compression rate (n/min): 100 vs 44 Sufficient rate (90-120/min): 91% vs 3% Correct hand position (%): 97% vs 75%
Neset A et al (2010) Norway [42]	RCT	(A) chest compression-only CPR (CCC) with feedback (n = 16) (B) 30:2 with feedback (n = 16) (C) CCC without feedback (n = 16) (D) 30:2 without feedback (n = 16)	Data from Skillreporter manikin and survey		A. CCC [(A)+(C)] vs 30:2 [(B)+(D)] Mean depth (mm): 41vs 42 (*p* = 0.14) Median number of compressions: 948 vs 574 (*p*<0.0005) Mean compression rate (/min): 96 vs 91 (*p* = 0.31) Total hands-off time (s): 2 vs 204 (*p*<0.0005) Mean ventilation rate (/min): N/A vs 4 Mean ventilation (ml): N/A vs 960 B. Feedback [(A)+(B)] VS No feedback [(C)+(D)] Mean depth (mm): 41 vs 42 (*p* = 0.27) Median number of compressions: 817 vs 705 (*p* = 0.010) Mean compression rate (/min): 101 vs 86 (*p* = 0.002) Total hands-off time (s): 195 vs 210 (*p* = 0.65) Mean ventilation rate (/min): 4 vs 2 (*p* = 0.026) Mean ventilation volume (ml): 902 vs 1395 (*p* = 0.009)
Nishiyama C et al (2010) Japan [32]	RCT	(A) chest compression-only CPR group (n = 106) (B) conventional CPR (30:2) group (n = 107)	Data from Skillreporter manikin		Group A vs Group B: Proportion of chest compressions with appropriate depth during 20-s CPR period Significant mean difference between 2 groups in 61–80 seconds (58.2% vs 74.3%, *p* = 0.003) Number of chest compressions: Group A>Group in any stage Mean no-flow time: 32.0 vs 81.0 (*p*<0.001) Mean time to first resuscitation (either chest compression or ventilation) (s): 32.0 vs 35.0 (*p* = 0.005)
Ghuysen A et al (2011) Belgium [29]	RCT	(A) untrained non-guided group (n = 30) (B) untrained guided group (by phone)(n = 30) (C) trained non-guided group (n = 25) (D) trained guided group (by phone) (n = 25)	Cardiff evaluation test and data from Skillreporter manikin	Group A vs Group B Airway management successful rate: 0% vs 57% (*p*<0.0001) Mean time to start of compressions (min): 0.27 vs 2.48 (P<0.0001) CPR performance score: 1.3 vs 4.8 (*p*<0.0001)	Group A vs Group B Mean depth (mm): 32 vs 41.5 Compression rate (n/min): 41.8 vs 59.7 (*p*<0.0001) Correct hand position: 16.7% vs 40% *The outcomes we compared here are between group (A) and group (B), excluding group (C) and (D) due to their nursing background.
Lee JS et al (2011) Korea [50]	RCT	(A) video group: received aid by watching a video on a cellular phone while performing compression-only CPR (n = 39) (B) audio group: had the aid of a voice dispatcher while performing compression-only CPR (n = 39)	Video reviewed by two emergency physicians and data from Skillreporter manikin	Group A vs Group B Total hands-off time: 11 vs 24 (non-significant) Percentage of no “hands-off” event after starting CPR: 71.8% vs 46.2% (*p*<0.05) Time to start of compressions (sec): 184 vs 211 (*p*<0.01) Correct hand position: 71.8% vs 43.6% (*p* = 0.01)	Group A vs Group B Mean depth (mm): 27.5 vs 31.3 (non-significant) Percentage of adequate compression depth: 20.5% vs 17.9% (non-significant) Mean compression rate (/min): 99.5 vs 77.4 (*p*<0.01) Percentage of adequate compression rate: 59% vs 28.2% (*p*<0.01)
Paal P et al (2012) Italy [43]	RCT	(A) assisted BLS group: with the aid of a BLS software program on a mobile phone (n = 64) (B) non-assisted BLS group: without the aid (n = 77)	Skillreporter manikin and a score chart.	Group A vs Group B Overall score (mean): 19.2 vs 12.9 (*p*<0.001) *Secondary endpoint: Check environment: 64% vs 27% (*p*<0.001) Protect from environmental risks: 70% vs 39% (*p*<0.001) Call for help: 56% vs 27% (*p*<0.001) Open the upper airway: 78% vs 16% (*p*<0.001)	Group A vs Group B Depth: non-significant Correct chest compression rate: 44% vs 14% (*p*<0.001) Ventilation: non-significant Mean time to start of compressions (sec): 165.3 vs 87.1 (*p*<0.001) Correct hand position: non-significant
Rössler B et al (2013) Austria [51]	RCT	(A) non-flowchart group: performed CPR without flowchart support (n = 41) (B) flowchart group: performed CPR with flowchart support (n = 43)	Evaluated by an independent investigator using a Skillreporter manikin	Group A vs Group B Completeness of BLS algorithm correctly (%): 0% vs 62% (*p*<0.0001) Feeling confidence 5 vs 7 (*p* = 0.0009) Fear of harming patients or making a mistake: non-significant	Group A vs Group B Mean depth (mm): 41 vs 43 (*p* = 0.49) Total number of compression (mean): 189 vs 200 (*p* = 0.55) Mean compression rate (/min): 76 vs 78 (*p* = 0.75) Mean compressions per cycle: 17 vs 28 (P<0.0001) Mean overall hands-off time (s): 169 vs 147 (*p* = 0.024) Corrected hands-off time (s): 146 vs 87 (*p*<0.0001) Mean time to start of compressions (sec): 23 vs 60 (*p*<0.0001)
Birkenes TS et al (2013) Norway [41]	RCT	(A) reference instruction group: based on ERC recommendations (n = 19) (B) intervention instruction group: using arm and nipple line (n = 18)	Measured using the laser beam at the upper and lower borders of the compressing hands and photographed	Correct hand position: Less caudal hand placement and the difference in mean hand position offset was 47 mm in intervention group (*p* = 0.001) None in the intervention group placed their hands in the abdominal region vs. 27.8% in the control group (*p* = 0.045)	
Buléon C et al (2013) France [45]	Randomized crossover controlled trial	(A) guided group: feedback by the CPRmeter device (n = 154) (B) blinded group: without feedback by the CPRmeter device (n = 154)	Data recorded by the CPR meter on a memory microSD card.		Group A vs Group B Mean depth (mm): 44:36 (*p*<0.001) Percentage of adequate depth: 85% vs 43% (*p*<0.001) Mean compression rate(/min): 107: 107 (non-significant) Percentage of adequate rate of chest compressions (CCs): 81% vs 56% (*p*<0.0001) Percentage of adequate release after CC: 100%:99% (non-significant) Rate of efficient compression (%): 71% vs 26% (*p*<0.0001) (Primary)
Eisenberg Chavez D et al (2013) USA [52]	RCT	(A) no dispatch instruction to remove clothing (n = 47) (B) dispatch instruction to remove clothing (n = 52)	Measured by study coordinator and data from Skillreporter manikin.	Group A vs Group B Mean time to first chest compressions (sec): 79 vs 109 (*p*<0.001)	Group A vs Group B Mean depth (mm): 40 vs 41 (*p*>0.05) Mean compression rate (/min): 99 vs 97 (*p*>0.05) Complete chest recoil: 91% vs 95% (*p*>0.05)
Park SO et al (2013) Korea [21]	RCT	(A) metronome group: metronome sounds played to the rescuer through the speaker (n = 35) (B) control group: without metronome sounds, substituted with repeat verbal encouragement (n = 35)	Data from Skillreporter manikin		Group A vs Group B Mean compression depth (mm): 45.9 vs 46.8 (*p* = 0.692) Median percentage of compression depth <38 mm: 69.2% vs 15.7% (*p* = 0.035) Median percentage of compression depth >51 mm: 13.5 vs 27.2 (*p* = 0.308) Mean compression rate (/min): 111.9 vs 96.7 (*p* = 0.018) Providers of correct compression rate: 32% vs 5% (*p*<0.0001) Mean numbers of chest compressions (/min): 109.4 vs 95.9 (P = 0.048) Mean total numbers of chest compressions: 439.4 vs 385.1 (*p* = 0.040) Median percentage of incomplete chest release: 0.0 vs 0.0 (*p* = 0.478) Mean time to start of compressions (sec): 35 vs 37 (*p* = 0.658) Median percentage of abnormal hand positions: 2.7 vs 22.7 (*p* = 0.361)
Birkenes TS et al (2014) Norway [27]	RCT	(A) standard T-CPR group: the rescuer perform CPR most of the time without dispatcher involvement (n = 49) (B) continuous T-CPR group: New protocol with some added instructions with speakerphone activation, removing obstacles and continuous instruction during CPR (n = 46)	Data from computer recorded manikin. Audio and video recordings reviewed by one person for time intervals.	Group A vs Group B Median time to first chest compression (sec): 84 vs 144 (*p*<0.001)	Group A vs Group B Mean absolute depth (mm): 48 vs 47 (*p* = 0.83) Depth≧40 mm (percentage of total compression)(median): 89 vs 80 (*p* = 0.83) Median total compressions: 870 vs 1000 (*p* = 0.014) Percentage of correct compression rate (90-120/min): 60% vs 87% (*p*<0.001) Mean compression rate (n/min): 108 vs 106 (*p* = 0.41) Median Hands-off time (s): 64 vs 12 (*p*<0.001) Correct hand position (%)(median): 99.7% vs 100% (*p* = 0.001)
Painter I et al (2014) USA [40]	RCT	(1) simplified scripts group (n = 39) (2) conventional scripts group (n = 36)	All data other than time to first compression were obtained by Skillreporter manikin.	Group A vs Group B Mean time to first compressions (sec): 99 vs 123 (*p*<0.01)	Group A vs Group B Mean compression depth (mm): 32 vs 25 (*p*<0.05) Percentage of compressions≧38 mm: 33% vs 20% (*p* = 0.14) Mean compression rate(/min): 102 vs 93 (*p* = 0.34) Mean percentage of sufficient rate: 24% vs 19% (*p* = 0.45) Mean compression fraction: 78 vs 77 (*p* = 0.78) Percentage of complete chest recoil: 89 vs 92 (*p* = 0.62) Mean number of hands-off periods: 5.3 vs 5.4 (*p* = 0.95) Mean total hands off time (s): 39 vs 41 (*p* = 0.78) Mean percentage of correct hand position: 63% vs 86% (*p*<0.01)
Rodriguez SA et al (2014) USA [35]	RCT	(1) Push hard group: Given “push as hard as you can” instructions (n = 64) (2) Two inches group: Given “push down approximately 2 inches” instructions (n = 64)	Data measured by a CPR recording defibrillator		Group A vs Group B Mean compression Depth (mm): 43 vs 36 (*p*<0.01) Percentage of adequate depth (≧ 47 mm): 39% vs 20% (*p* = 0.02) Mean compression rate (/min): 93 vs 82 (*p* = 0.06) Percentage of adequate rate (≧ 100/min): 36% vs 30% (p = 0.45) Percentage of subjects achieving full chest recoil: 53% vs 75% (*p* = 0.01)
van Tulder R et al (2014) Austria [26]	RCT	(A) standard instruction group:”push down firmly 5cm” (n = 8) (B) repeated standard instruction group: repeating the instruction every 20 s (n = 8) (C) intensified wording group: “It is very important to push down the chest firmly 5 cm every time” (n = 8) (D) repeated intensified wording group (n = 8)	Data from simulator manikin		Group A vs Group B Mean compression depth (mm): 43 vs 32 vs 20 vs 22 Mean compression rate (/min): 93 vs 89 vs 93 vs 101 Leaning depth: 8 vs 7 vs 5 vs 8 Mean cumulative hands off (s): 60 vs 134 vs 157 vs 146 Time to start of compressions (sec): 52 vs 50 vs 47 vs 60
Kim YH et al (2015) Korea [47]	RCT	(A) same side group: two rescuers on the same side (n = 32) (B) opposite side group: two rescuers on the opposite side (n = 32)	Data from Skillreporter manikin		Group A vs Group B Mean depth (mm): 38 vs 37 (*p* = 0.616) Total number of compressions (median): 815 vs 811 (*p* = 0.381) Percentage of adequate compression (median): 2.5% vs 1.0% (*p* = 0.171) Percentage of incomplete chest recoil (median): 16 vs 4 (*p* = 0.564) Cumulative hand-off time (s)(mean): 6.6 vs 4.5 (*p* = 0.005) Percentage of incorrect hand position (median): 11% vs 19% (*p* = 0.361)
Rasmussen SE et al (2017) Denmark [33]	RCT	(A) novel protocol group: designed based on previous research and pilot studies (n = 61) (B) standard protocol (n = 64)	Data was sampled from the manikin. Video recordings were assessed independently by two ERC certified BLS/AED instructors.	Group A vs Group B Overall score (points)(mean): 18.6 vs 17.5 (*p*<0.001)	Group A vs Group B Mean compression depth (mm): 58 vs 52 (*p* = 0.02) Mean compression rate (/min): 114 vs 110 (*p* = 0.04) Compressions without total recoil (%)(median): 14 vs 8 (*p* = 0.06) Hands-off time per min (s)(median): 6 vs 1 (*p*<0.001) Time to start of compressions (sec)(median): 65 vs 72 (*p*<0.001) Correct hand position (%): 61% vs 23% (*p* = 0.01)
Sakai T et al (2015) Japan [19]	RCT	(A) CPR support application group: with the aid of the CPR support application on a smartphone (n = 43) (B) control group: without the aid of the CPR support application on a smartphone (n = 41)	Data from Skillreporting manikin		Group A vs Group B Mean compression depth (mm)(mean): 35.0 vs 36.7 (*p* = 0.492) Number of chest compressions with appropriate depth (mean): 65.7 vs 41.0 (*p* = 0.095) Time without chest compression (s)(mean): 4.4 vs 63.8 (*p*<0.001) Mean time to start of compressions or ventilations (s): 37.1 vs 29.3 (*p* = 0.048) Number of chest compressions with correct hand position (mean): 109 vs 42.6 (*p*<0.001) Rate of calling 119: 67.4% vs 46.3% (*p* = 0.041) Rate of requesting AED: 60.5%: 22.0% (*p*<0.001) Chest compressions performed: 100% vs 75.6% (*p*<0.001) Number of total chest compressions (mean): 211.6 vs 77.0 (*P*<0.001)
Krikscionaitiene A et al (2016) Lithuania [30]	RCT	(A) control group: Standard hands-only CPR with two-hands chest compression (n = 32) (B) intervention group: hands-only CPR with Andrew’s manoeuver (four-hands chest compression) (n = 34)	Data from Skillreporter manikin		Group A vs Group B Mean compression depth (mm): 47.8 vs 54.2 (*p* = 0.002) Number of chest compressions with adequate depth (5-6cm) (mean): 188 vs 334 (*p* = 0.012) Percentage of chest compression with adequate depth: 46.9% vs 74.8% (*p* = 0.003) Number of total chest compressions (mean): 394 vs 444 (*p* = 0.831) Mean chest compression rate (/min)(mean): 97.3 vs 91.0 (*p* = 0.352) Percentage of leaning (mean):1.1% vs 0.8% (*p* = 0.639) Mean chest compression fraction: 85.6% vs 86% (*p* = 0.882) Mean hand-off time (sec): 69.1 vs 67.2 (*p* = 0.882) Mean percentage of chest compression duty cycle: 40.8% vs 44 (*p* = 0.083)
Spelten O et al (2016) Germany [31]	RCT	(A) U-CPR group: uninstructed CPR (n = 20) (B) DACO-CPR group: dispatcher-assisted compression-only CPR (n = 19) (C) DAF-CPR group: full dispatcher-assisted CPR including rescue ventilation (n = 19)	Manikin and software. Hand positioning and head-tilt for ventilation reviewed by two independent investigators via video recordings.		Group A vs Group B vs Group C Mean compression depth (mm): 40.6 vs 41.0 vs 38.8 (*p*>0.05) Mean compression rate (1/min): 35.6 vs 65.5 vs 44.5 (*p* = 0.001) Percentage of compressions without correct release(mean): 13.2% vs 16.9% vs 6.5% (*p*>0.05) Overall no-flow-time(sec)(mean): 273.4 vs 99.8 vs 240.1 (p<0.001) Total number of ventilation attempts (mean): 37.44 vs 2.0 vs 23.26 (group A: C: *p* = 0.006) Time to start of compressions (sec)(mean): 25.1 vs 55.2 vs 101.2 (group B:C:*p*<0.001) Percentage of compressions with wrong hand position (mean): 15.6% vs10.5% vs 16.0% (*p*>0.05) Numbers of total compressions (mean): 293.53 vs 512.11 vs 356.53 (*p* = 0.001)
Stipulante S et al (2016) Belgium [39]	RCT	(A) t-CPR group: only receiving audio telephone instructions (n = 60) (B) v-CPR group: receiving videoconferencing and perform CPR (n = 60)	Audio-video recordings evaluated by investigators and Skillreporter manikin.	Open the airway successfully: 68% vs 98.3% (*p*<0.0001) Breathing check: 82% vs 98.3% (*p* = 0.003) Time for responsiveness check (s)(median): 34.5 vs 39 (*p* = 0.0043) Time for airway opening (s)(median): 72 vs 66.5 (*p* = 0.18) Time for breathing check (s): 85 vs 93.5 (*p* = 0.08)	Group A vs Group B Mean compression depth (mm): 47.1 vs 48.38 (*p* = 0.64) Percentage of compression with appropriate depth: 40.3% vs 43.3% (*p* = 0.85) Mean Compression rate (/min): 85.6 vs 110.4 (*p*<0.0001) Total number of chest compressions (median): 301 vs 421 (*p*<0.0001) Percentage of compressions with appropriate rate: 37.9% vs 80% (*p*<0.0001) Hands-off time (s)(median): 7 vs 0 (*p*<0.0001) Time to start of compressions (sec)(median): 122.5 vs 146 (*p*<0.0001) Percentage of compressions with correct hand positioning: 68% vs 91.7% (*p* = 0.0017)
Torney H et al (2016) UK (Experiment 2) [38]	RCT	(A) CPR rate feedback group (n = 68) (B) control group: without CPR rate feedback group (n = 72)	Data from public access defibrillator		Group A vs Group B Mean compression depth (mm): 24.61 vs 20.08 (p = 0.001) Percentage of participants achieving good CPR compression speed within 45s: 95.6% vs 62.5% (*p*<0.0001) Mean percentage chest compression fraction: 91.6% vs 88.7% (non-significant)
Hurst V 4^th^ et al (2007) USA [53]	Crossover interventional study	(A) BVM group: bag-valve mask with self-inflating bag (n = 40) (B) Model 730 group: a pneumatically powered transport ventilator that is specifically developed for field use by personnel who have a wide range of training and expertise (n = 40)	Data were collected on a laptop computer using devices and software from the research pneumotach system.		Group A vs Group B Mean number of compressions (4 min cycle): 281.85 vs 230.75 (*p*<0.05) Mean number of breaths (4 min cycle): 38.1 vs 32.0 (*p*<0.05) Mean delivered tidal volume per breath (ml): 803.03 vs 672.08 (*p*<0.05) Mean delivered airway flow rate per breath (ml/min-breath): 161.01 vs 21.31 (*p*<0.05) Mean delivered airway pressure per breath (cmH2O): 14.43 vs 7.54 (*p*<0.05)
Atkinson PR et al (1999) UK [17]	RCT	(A) CPR with no additional instruction (n = 9) (B) CPR with receiving telephone instruction (n = 10) (C) CPR with advice over a video-link (n = 10) (D) CPR with advice by an instructor standing beside them (n = 9)	The CPR standard was determined by 2 observers and by computerized analysis of manikin recordings.	Group A vs Group B vs Group C vs Group D Total number of correct ventilations (median): 0 vs 8.5 vs 2.5 vs 2 (compared with group A, the other groups were all significantly difference.)	Group A vs Group B vs Group C vs Group D Total numbers of correct chest compressions (median): 0 vs 0.5 vs 7.5 vs 10 (Group A: Group B, *p* = 0.11; Group A: Group C, *p* = 0.021; Group A: Group D, *p* = 0.046) Time to onset of CPR (sec)(median): 7 vs 30 vs 35 vs 34 (Compared with group A, the other groups were all significantly different.) Total numbers of compressions with correct hand position but incorrect depth (median): 2 vs 16.5 vs 35 vs 43(Group A: Group B, *p* = 0.23; Group A: Group C, *p* = 0.023; Group A: Group D, *p* = 0.002)
Liu S et al (2016) Canada [18]	Randomized crossover trial	(A) CCC (continuous chest compression) group (n = 63) (B) 30:2 group (n = 62)	Recordings from manikin		Group A vs Group B Numbers of adequate chest compressions depth (≧5cm)(mean): 381.5 vs 324.9 (*p* = 0.0001) Numbers of adequate chest compressions decreased over time in CCC group (*p*<0.0001) but not 30:2 group (p = 0.75) Mean number of chest compressions (mean): 480 vs 376.3 (*p*<0.0001) Mean compression rate (/min): 99.7 vs 101.8 (*p* = 0.0002)
Trenkamp RH et al (2015) USA [24]	Observational study	(A) manual group: performing CPR with their hands (n = 49) (B) heel group: performing CPR with their heels (n = 49)	Recordings from the manikin		Group A vs Group B Percentage of performing compliant compressions for 10 minutes: 16% vs 65% Percentage of subjects performing compressions without adequate depth: 24% vs 2% Length of time to perform compliant compressions (sec)(mean): 2.9 vs 7.9 (*p*<0.001)
Birkenes TS et al (2012) Norway [48]	Observational study	intervention: continuous telephone-instructed 30:2 CPR with duration of 10 minutes. Compare CPR performance within first minute with those within 10^th^ minute.	Video recordings reviewed by researchers and recordings from the manikin	1^st^ min VS 10^th^ min Chin lift: 17/29 vs 18/29 (*p* = 1.0) Head lift: 14/29 vs 20/29 (*p* = 0.15) Nose pinch: 19/29 vs 22/29 (*p* = 0.25) Hand placement on nipple line: 17/29 vs 24/29 (*p* = 0.065) Participants communicating with dispatcher and performing CPR simultaneously): 29/30 vs 29/30 (non-significant) Correct rescuer position for chest compressions: 13/30 vs 21/30 (*p* = 0.008)	1^st^ min VS 10^th^ min Mean compression depth (mm): 43 vs 42 (non-significant) Mean compression rate (/min): 84 vs 101 (*p*<0.001) Mean time between compression series (s): 20.5 vs 12.1 (*p*<0.001) Percentage of participants achieving successful ventilations: 13/30 vs 23/30 (*p* = 0.006)
White AE et al (2017) Singapore [54]	Randomized crossover controlled study	(A) chest compression with CPRcard feedback (n = 35) (B) chest compression without CPRcard feedback (n = 35)	CPRcard or Resusci Anne’s SimPad SkillReporter		Group A vs Group B Mean compression depth (cm) (median): 5.0: 5.0 (*p* = 0.319) Mean compression rate (/min) (median): 117: 122 (*p* = 0.001) Adequate compression rate (median) 83%: 47% (*p*< 0.001) Adequate depth (median) 52%: 48% (*p* = 0.957) Met compression rate of 100–120/min & depth ≥ 5 cm(n,%) 9 (36%): 1 (4%) (*p* = 0.022)
Wutzler A et al (2018) Germany [55]	Randomized crossover controlled study	(A) chest compression with audiovisual feedback (n = 48) (B) chest compression without audiovisual feedback (n = 48)	Data from Physio-Control (TrueCPR Report Generator)		Group A vs Group B Mean compression depth (mm): 54: 55.6 (*p* = 0.789) Mean compression rate(/min): 98.4: 95.7 (*p* = 0.937) Percentage of optimal chest compression(%)(mean) 58.9:14.6 (*p* < 0.0001) Longest interval without optimal chest compression (sec) (mean) 27.5: 76.5 (*p* < 0.0001) Effective chest compression trials (%) 45.8: 0 (*p* < 0.0001)
Liu Y et al (2018) China [56]	RCT	(A) hands-only CPR (AHA 2010 guidelines) without feedback (n = 42) (B) hands-only CPR (AHA 2015 guidelines) without feedback (n = 42) (C) hands-only CPR (AHA 2015 guidelines) with feedback (n = 40)	Data from LinkCPR (SunLife, China)		Group A vs Group B vs Group C Mean compression depth (mm) 1 min: 49: 51: 56 (*p* <0.05) 2 min: 44: 49: 56 (*p* <0.05) Mean compression rate (/min)(mean) 1 min: 118: 112: 104 (*p* <0.05) 2 min: 115: 109: 104 (*p* <0.05) Percentage of correct chest compression depth (%)(mean) 1 min: 63.1: 37.6: 89.1 (*p* <0.05) 2 min: 64.2: 35.8: 88.4 (*p* <0.05) Percentage of correct chest compression rate (%)(mean) 1 min 83.5: 61.9: 86.5 (*p* <0.05) 2 min: 76.4: 58.9: 85.9 (*p* <0.05) Percentage of correct chest compression (%)(mean) 1 min: 54.9: 29.6: 87.8 (*p* <0.05) 2 min: 53.6: 25.6: 87.1 (*p* <0.05)
Eaton G et al (2018) UK [57]	Randomized crossover study	(A) CPR with PocketCPR (n = 118) (B) CPR without PocketCPR (n = 118)	Data from the manikin software (Laerdal Resuscitation manikin)		Group A vs Group B Percentage of mean correct compression depth (5-6mm): 44.28%: 40.57% (*p* = 0.001) Mean Compression rate (/min)(mean) 106.87: 105.37 (*p* = 0.858) Mean total compression (/2min)(): 205.19: 163.25 (*p* < 0.001) Time to start of compressions (sec): Nil Correct hand position: non-significant
Scott G et al (2018) USA [58]	RCT	(A) CPR under dispatcher’s instruction with the use of the metronome tool (n = 85) (B) CPR under dispatcher’s instruction without the use of the metronome tool (n = 63)	Data from simulator manikin		Group A vs Group B Correct compression depth (5-6mm), n (%) 4 (4.7%): 2 (3.2%) Correct compression rate: achieving target rate (100-120/min), n (%) 39 (45.9%): 14 (22.2%) (*p* = 0.003)

All RCTs had a high risk of bias in blinding of the participants and personnel. Blinding of outcome assessment was rated high risk in 13 studies because the measurement included video recording review [17,18,20,23,27–29,33,39,43,50–52]. Among non-RCT studies, 3 studies earned full points of 9 [24,49,53]. Tables 2 and 3 show the risk of bias for included studies.

**Table 2:** Risk of bias for included studies.

First author (year published)	Study design	Random sequence generation	Allocation concealment	Blinding of participants and personnel	Blinding of outcome assessment	Incomplete outcome data	Selective reporting	Other sources
Woollard M et al (2003) [25]	RCT^a^	L	U	H	U	L	L	L
Williams JG et al (2006) [44]	RCT	U	U	H	L	L	L	L
Dias JA et al (2007) [34]	RCT	L	U	H	L	L	L	L
Brown TB et al (2008) [22]	RCT	L	U	H	L	L	L	L
Mirza M et al (2008) [37]	RCT	L	U	H	L	L	L	L
Nikandish R et al (2008) [36]	Randomized crossover controlled trial	L	L	H	L	L	L	L
Yang CW et al (2008) [28]	RCT	U	U	H	H	L	L	L
Bolle SR et al (2009) [46]	RCT	U	U	H	L	L	L	L
Yang CW et al (2009) [20]	RCT	L	U	H	H	L	L	L
Merchant RM et al (2010) [23]	RCT	L	H	H	H	L	L	L
Neset A et al (2010) [42]	RCT	U	U	H	L	L	L	L
Nishiyama C et al (2010) [32]	RCT	U	U	H	L	L	L	L
Ghuysen A et al (2011) [29]	RCT	L	L	H	H	L	L	L
Lee JS et al (2011) [50]	RCT	L	H	H	H	L	L	L
Paal P et al (2012) [43]	RCT	L	U	H	H	L	L	L
Rössler B et al (2013) [51]	RCT	L	L	H	H	L	L	L
Birkenes TS et al (2013) [41]	RCT	L	L	H	L	L	L	L
Buléon C et al (2013) [45]	Randomized crossover controlled trial	L	L	H	L	L	L	L
Eisenberg Chavez D et al (2013) [52]	RCT	L	L	H	H	L	L	L
Park SO et al (2013) [21]	RCT	L	L	H	L	L	L	L
Birkenes TS et al (2014) [27]	RCT	L	L	H	H	L	L	L
Painter I et al (2014) USA [40]	RCT	L	L	H	U	L	L	L
Rodriguez SA et al (2014) [35]	RCT	U	U	H	L	L	L	L
van Tulder R et al (2014) [26]	RCT	L	L	H	L	L	L	L
Kim YH et al (2015) [47]	RCT	L	U	H	L	L	L	L
Rasmussen SE et al (2017) [33]	RCT	L	H	H	H	L	L	L
Sakai T et al (2015) [19]	RCT	L	U	H	L	L	L	L
Krikscionaitiene A et al (2016) [30]	RCT	L	L	H	L	L	L	L
Spelten O et al (2016) [31]	RCT	L	L	H	L	L	L	L
Stipulante S et al (2016) [39]	RCT	L	L	H	H	L	L	L
Torney H et al (2016) [38]	RCT	U	U	H	L	L	L	L
Atkinson PR et al (1999) [17]	RCT	U	U	H	H	L	L	L
Liu S et al (2016) [18]	Randomized crossover controlled trial	U	L	H	H	L	L	L
White AE et al (2017) [54]	Randomized crossover controlled trial	U	L	H	L	H	L	L
Wutzler A et al (2018) [55]	Randomized crossover controlled trial	L	L	H	L	L	L	L
Liu Y et al (2018) [56]	RCT	U	U	H	L	L	H	L
Eaton G et al (2018) [57]	Randomized crossover study	L	L	H	L	L	L	L
Scott G et al (2018) [58]	RCT	L	U	H	L	L	L	L

^a^RCT: randomized control trial.

**Table 3:** Quality assessment using Newcastle-Ottawa Scale.

Study	Representativeness of exposed cohort	Selection of the non exposed cohort	Ascertainment of exposure	outcome of interest was not present at start of study	Comparability of cohorts	Assessment of outcomes	Follow-up long enough for outcomes to occur	Adequacy of follow up of cohorts	Total
Kellermann AL et al (1989)[49]	1	1	1	1	2	1	1	1	9
Hurst V 4^th^ et al (2007) [53]	1	1	1	1	2	1	1	1	9
Trenkamp RH et al (2015) [24]	1	1	1	1	2	1	1	1	9
Birkenes TS et al (2012) [48]	1	1	1	1	1	1	1	1	8

### Results of the individual studies

We grouped the interventions of studies included into three groups: (A) modifications to dispatcher-assisted CPR (DA-CPR), (B) Different methods to perform CPR, and (C) additional aids to bystander CPR. The summary of interventions in studies included is shown in Table 4.

**Table 4:** Summary of interventions for the quality of bystander cardiopulmonary resuscitation in the included studies.

Modifications to DA-CPR	
Modified telephone DA-CPR instructions	
Added instructions with speakerphone activation, removing obstacles and continuous instruction [27]	Improved CPR quality, but longer time to first chest compression
Simplified compression only-CPR protocols [34,40]	Improved CPR quality and shorter time to first chest compression
Instructions with“push as hard as you can” [37]	Improved compression depth compared to instruction with “push down firmly 2 inches”
Modified instructions using arm and nipple line [41]	Improved hand position
Elimination of the instruction to remove the victim’s clothing [52]	Shortened time to first compression without affecting CPR quality
Novel protocols with changing instructional content of hand position, compression depth and compression rate at the same time [33]	Improved CPR quality compared to standard protocol
Added instructions with “put the phone down” [22]	Similar CPR quality compared to instruction without “put the phone down”
Instructions with repeated or intensified wording to remind of or emphasize the importance of chest compression depth [26]	Similar CPR quality compared to standard instruction
Instructions with “push as hard as you can” for paediatric CPR[35]	Similar CPR quality compared to instruction with “push down approximately 2 inches”
Video-assisted DA-CPR	
Video-conferencing DA-CPR [17,20,28,39,46]	Improved CPR quality in 4 studies [17,20,28,39]; no improvement in 1 study compared to telephone CPR [46] Longer time to first chest compression/rescuer breathing in 3 studies [20,28,39]; similar time to first chest compression [17,46] compared to telephone CPR
Showing a video on cellular phone when performing CPR [50]	Improved CPR quality and shorter time to first chest compression compared to telephone CPR
Playing metronome sounds to the rescuer by the dispatcher [21, 58]	Improved chest compression rates, but tended to shallow compressions
Different methods of performing CPR	
Compression-only CPR [18,25,32,42,44]	Improved CPR quality compared to conventional CPR in 5 studies [18,25,32,42,44] Easy physical fatigue in 2 studies [18,32], but similar fatigue in 3 studies [25,42,44] compared to conventional CPR
Dominant hand against the chest wall [36]	Similar CPR quality when compared to non-dominant hand against the chest wall
Two rescuers on the opposite sides [47]	Reduced hands-off time compared to two rescuers at the same side
Four-hand CPR [30]	Improved chest compression depth compared to two-hand CPR
CPR with heels [24]	Improved chest compression depth compared to CPR with hands
Additional aids to bystander CPR	
Telephone DA-CPR [23,29,31,48,49]	Improved CPR quality compared to no telephone DA-CPR
Simple basic life support flowchart [51]	Improved CPR quality compared to no such flowchart
Basic life support software programs on a mobile phone with a metronome function [43]	Improved CPR quality, but longer time to first chest compression compared to no such software program.
Newly-developed CPR support applications on a mobile phone [19]	Increased number of total chest compressions, but longer time to start compressions or ventilations compared to no such application
Real-time feedback devices [38,42,45,54–57]	Improved CPR quality compared to no such application.
Pneumatically powered transport ventilators [53]	Improved ventilation quality compared to bag-valve mask

CPR: cardiopulmonary resuscitation; DA-CPR: dispatcher-assisted cardiopulmonary resuscitation.

#### (A) Modifications to dispatcher-assisted CPR

There were seventeen studies where the intervention was related to modifications to DA-CPR [17,20–22,26–28,33–35,37,39–41,46,50,52]. The interventions are described below.

**Modified telephone DA-CPR instructions**. There were ten studies that employed interventions of modified telephone DA-CPR instructions [22,26,27,33–35,37,40,41,52]. Among them, seven studies found that the interventions improved the quality of CPR or shorten time to start chest compressions [27,33,34,37,40,41,52]. These interventions included adding instructions with speakerphone activation, removing obstacles, and continuous instruction during CPR [27], simplified compressions-only CPR instructions [34,40], instruction to “push hard as you can” for adult with cardiac arrest [37], modified instruction using arm and nipple line [41], eliminating the instruction to remove the victim’s clothing [52], and a novel instructional protocol with changing instructional content of hand position, compression depth and compression rate at the same time [33].

The other three studies showed no significant improvement in the quality of CPR, including the added instruction of “put the phone down” [22], repeated or intensified wording to remind or emphasize the importance of chest compression depth [26], and for paediatric CPR using “push as hard as you can.” [35]

**Video-assisted DA-CPR**. Six studies discussed whether video-assisted DA-CPR improved CPR quality when compared to telephone DA-CPR [17,20,28,39,46,50]. Among those, five studies used video-conferencing DA-CPR [17,20,28,39,46] and one study asked the participants to watch a video on cellular phone when performing CPR [50]. The comparison between video-assisted and telephone DA-CPR for different components of CPR quality in studies is shown in Table 5.

**Table 5:** The comparison between video-assisted and telephone DA-CPR for different components of CPR quality in 6 studies.

	Correct chest compression depth	Correct chest compression rate	Full chest recoil	Minimal interrupted chest compression	Correct hand position	Shorter time to start chest compression	Ventilation
Number of studies showing video-assisted DA-CPR is superior	2^17,20^	4^17,20,39,50^	0	1^39^	3^17,39,50^	1^50^	2^28,39^
Number of studies showing telephone DA-CPR is superior	0	0	0	2^20,50^	0	3^20,28,39^	1^17^
Number of studies showing both methods were the same	3^39,46,50^	1^46^	1^46^	1^46^	2^20,46^	2^17,46^	1^46^
Number of studies without measurement	1^28^	1^28^	5^17,20,28,39,50^	2^17,28^	1^28^	0	1^20^

The conclusions of five studies were in favor of video-assisted DA-CPR [17,20,28,39,50]. Only one study demonstrated video communication was unlikely to improve telephone CPR significantly [46]. However, three studies showed that video-conferencing DA-CPR has increased time to first chest compression/rescuer breathing when compared to audio-assisted DA-CPR [20,28,39]. The other two studies had the opposite result, showing that video-conferencing DA-CPR did not have significantly longer time to first chest compression [17,46]. One study in which the participants watched a video on cellular phone during CPR had a shorter time starting chest compression when compared with audio-assisted DA-CPR [50]. Among the six studies, video-assisted DA-CPR is superior to, at least equivalent to telephone DA-CPR on the performance of correct chest compression depth, correct chest compression rate and correct hand position (Table 5).

**Playing metronome sounds to the rescuer**. In one study, metronome sounds were played to the rescuers through their mobile phone when performing DA-CPR, and it showed no improvement in the overall CPR quality even though it improved the chest compression rates [21], but was associated more with shallow compressions than the conventional telephone dispatcher-assisted compression-only CPR. Another study showed the rescuers receiving emergency medical dispatchers’ instructions with metronome assistance performed better with correct compression rate than those receiving instructions without metronome assistance [58]. However, the compression depth tended to be shallower under metronome assistance.

#### (B) Different methods of performing CPR

There were nine studies comparing different methods of performing CPR [18,24,25,30,32,36,42,44,47].

**Compression-only CPR vs. conventional CPR**. There were five studies comparing the quality of CPR between compression-only CPR and conventional CPR with compression: breath ratio as 30:2 [18,25,32,42,44]. All of these studies showed that compression-only CPR had better CPR quality than the conventional CPR since compression-only CPR got more chest compressions, less hands-off time, and less time to first compression. However, two studies pointed out that chest compressions with appropriate depth decreased more rapidly in groups with compression-only CPR than those with conventional CPR due to increased physical fatigue [18,32]. A study performed in Japan even found that appropriate chest compression depth decreased significantly one minute after starting compression-only CPR [32]. On the other hand, two studies performed in the UK and Norway showed no difference in CPR-related exhaustion between the compression-only CPR group and the conventional CPR group during the 10-minute test [25,42], and one study performed in the United States also revealed no difference in perceived fatigue in both groups after performing CPR for 3-minutes [44].

**Dominant vs. non-dominant hands**. One study explored whether there were any differences in CPR quality when laypersons compressed the victim’s chest with their dominant or non-dominant hand against the chest wall [36]. This study demonstrated that, although there was a trend towards increased incidence of correct chest compressions when the dominant hand was positioned in contact with the sternum, it did not have statistical significance for a 5-minute-long CPR session.

**Opposite sides vs. the same side**. One study compared CPR quality between two rescuers being on the same side and being on the opposite sides in a two-rescuer situation [47]. The study showed that changing compression from the opposite sides reduced hands-off time compared to changing on the same side in prehospital hands-only CPR provided by two bystanders. The other parameters such as CPR quality were similar between the two groups.

**Four hands vs. two hands**. One study compared the quality of four-hand CPR to that of a two-hand CPR [30]. The study showed that four-hand chest compression during the simulated DA-CPR significantly improved the chest compression depth without affecting the compression rate among older female rescuers.

**Heels vs. hands**. One study compared the quality of CPR when chest compressions were applied with heels or hands [24]. The study results showed significantly more compressions meeting guidelines and fewer compressions without adequate depth in the heel group. The study concluded that heel compressions were useful in situations where a lone rescuer could not get down on the floor, could not compress the chest to adequate depth because of an infirmity or lack of weight, or when the rescuer became too tired to continue manual compressions.

#### (C) Additional aids to bystander CPR

There were twelve studies related to additional aids to bystander CPR [19,23,29,31,38,42,43,45,48,49,51,53].

**Telephone DA-CPR**. There were five studies including telephone DA-CPR intervention [23,29,31,48,49]. Three of them were RCTs [23,29,31]. All of them demonstrated telephone DA-CPR improved the overall quality of CPR. One study showed that dispatcher-assisted compression-only CPR had better CPR quality than dispatcher-assisted conventional 30:2 CPR [31]. Another non-RCT showed that participants with CPR training before receiving telephone instructions had better CPR performance than those without CPR training before receiving the same telephone instructions [49].

**Simple basic life support flowchart**. One study demonstrated that the quality of bystander CPR could be improved significantly by a simple basic life support (BLS) flowchart offered to the rescuer [51].

**Assistance via mobile phone**. One study demonstrated that the BLS software program on a mobile phone, which had a metronome function, contributed to a better overall performance [43]. Nevertheless, participants with the mobile program took a longer time to call the dispatch centre and to start chest compressions. Another study showed that the newly-developed CPR support application (app) for smartphones resulted in an increased number of total chest compressions performed [19]. However, the participants with the new app delayed starting compressions or ventilations.

**Assistance via real-time feedback device**. Certain kinds of real-time feedback devices for CPR performance improved the quality of bystander CPR in seven studies. One study showed that the group with visual real-time feedback from PC Skillreporter performed more compressions and had a higher rate of chest compression with less hands-off time when compared with those without such feedback [42]. One study revealed that the group with visual real-time feedback from CPR meter, a device put between the victim’s chest and the rescuer’s hands when performing CPR, significantly improved chest compression quality [45]. Another study showed that, with feedback on compression rate from the test device, the rescuer could perform higher quality of CPR with higher compression rate and without compromising compression depth [38]. The other 4 studies also found that real-time feedback devices improved the quality of bystander CPR in a simulation setting [54–57].

**Usage of M730 (a pneumatically powered transport ventilator)**. One study showed that M730 ventilator yielded better ventilation quality than bag-valve mask, resulting in lower delivered airway flow rate, lower airway pressure and lower volume of gas entering the stomach per breath [53].

## Discussion

Our review showed that telephone DA-CPR seemed to improve the overall quality of bystander CPR. Further studies revealed that telephone DA-CPR with simplified or more concrete instructional protocols might further improve the quality of bystander CPR. It suggested that more efforts might be needed in the future not only for dispatchers to identify patients with cardiac arrest and instruct the bystander to perform CPR, but also to build up an effective instructional protocol to let bystanders perform high-quality CPR. Including effective on-scene interventions into instructional protocols, such as four-hand CPR for elderly rescuers, kneel on opposite sides for two-person CPR, and CPR with heels for a tired rescuer, could improve the overall quality of CPR. In addition, it had been shown that, among participants who received the same telephone instruction, the participants receiving CPR training before had better CPR performance than those without receiving any CPR training [49]. It seemed that prior CPR education and telephone DA-CPR had a synergistic effect on the quality of bystander CPR. Therefore, to improve the survival of victims with SCA by improving the quality of bystander CPR, the importance of CPR education and the effective strategy of telephone DA-CPR should be emphasized to the community. More people who receive CPR training would improve the overall quality of bystander CPR. It could translate into better outcomes for patients with out-of-hospital cardiac arrest if an effective, evidence-based protocol of telephone DA-CPR is implemented in the community.

Although dispatcher-used video devices, including mobile phones, can be used to communicate with bystanders to improve some parameters of CPR quality, it is crucial to make the rescuer to perform CPR as fast as possible. Some studies revealed that video-assisted DA-CPR had more time starting chest compression or rescue breathing than audio-assisted DA-CPR. The increased non-flow time might compromise the clinical outcomes of patients with SCA. Further studies on video-assisted DA-CPR are needed to overcome this problem.

It is also worth noting that additional assistance from electronic devices has been shown to improve the quality of bystander CPR. Various electronic devices with apps and software are currently being developed. These devices could provide readily available instructions, sensing and feedback, which might improve the quality of bystander CPR. However, using such mobile devices also caused rescuers to delay starting chest compressions or ventilations in some studies [19,43]. An app or software on mobile devices with clear and easy-to-understand content that can be activated easily is helpful for rescuers to start CPR quickly.

Our study also revealed that compression-only CPR had better CPR quality and less time spent starting chest compression compared with conventional CPR. It has been shown that, by skipping the breathing part, compression-only CPR not only increased the compression rate of bystander CPR, but also improved the clinical outcomes of adult victims [59]. Additionally, one study showed that the quality of dispatcher-assisted compression-only CPR was better than that of dispatcher-assisted conventional CPR [31]. It was also recommended that dispatchers should provide compression-only CPR instructions to callers for adults with suspected out-of-hospital cardiac arrest by the guidelines of International Liaison Committee of Resuscitation, European Resuscitation Council and American Heart Association [6–8]. However, some studies pointed out the possibility of compression-only CPR exhausting the first rescuer quickly and compromising the quality of CPR before the medical personnel arrived [32,60]. In our review, there were different results among studies about whether compression-only CPR exhausted rescuers more quickly. One study showed that the quality of chest compressions rapidly declined in compression-only CPR when compared with performing conventional CPR for only one minute [32]. Another study involving elderly volunteers also showed that the conventional CPR group had significantly more adequate depth of chest compressions than the compression-only CPR group one minute after starting CPR [60]. However, other studies revealed no differences between the two methods [25,42,44]. The different results hinted that compression-only CPR might cause rescuers with less muscle strength, low body weight or older age to become fatigued quickly [61]. These types of rescuers may change their roles sooner than every 2 minutes to maintain the quality of chest compressions during compression-only CPR if there are two or more bystanders at the scene.

In our study, we only included studies whose participants were laypersons. Although medical personnel might also be at the scene of the event of a cardiac arrest and perform bystander CPR, most of the cardiac arrest events happened outside health care institutions, and it is reasonable to assume that bystander CPR was performed by laypersons in most cases. Hence, we only selected laypersons in our study. In addition, the interventions found to be effective in improving the quality of CPR performed by laypersons could reasonably be speculated to improve of the quality of CPR performed by the medical personnel.

From our review, strategies to improve the quality of bystander CPR were proposed as follows. Telephone DA-CPR with effective, evidence-based instructions to instruct callers to perform chest compression-only CPR for adults with SCA may be implemented first. If only one rescuer performs CPR on the scene and feels tired, CPR with heels may be suggested. If more than one rescuer performs CPR, a second rescuer is suggested on the opposite side of the first rescuer. The rescuers with less muscle strength, low body weight or older age may change their roles sooner than every 2 minutes. Four-hand CPR may be suggested to elderly rescuers. Real-time feedback devices are considered to be used during CPR if available. Easy-to-understand information about how to perform high-quality CPR via mobile devices may be provided to rescuers immediately. Finally, before dispatchers use video devices, such as mobile phones, to communicate to rescuers, a simplified communication protocol to minimize time to start resuscitation should be designed and proved effective first.

In our study, we did not perform meta-analysis due to high inconsistencies among included studies in study designs, interventions and types of data presentations. Therefore, our study cannot report any conclusive results and can only give a clue about that what interventions might be helpful in improving bystander CPR quality. Further evaluation will be needed before an intervention is implemented in a community.

## Limitations

There were some limitations in our study. First, during our search for studies on interventions which could improve the quality of bystander CPR, we could not find a study performed in a real-life resuscitation situation. Whether such interventions associated with higher quality of bystander CPR could be translated to better victims’ outcomes remained unknown. However, several studies have already shown that high-quality CPR could be translated to good outcomes for the victims [9–12]. Second, the inconsistencies in CPR quality measurement among studies were high. There were different parameters that were recorded in studies included, and how the researchers measured CPR quality was also different. In addition, there were inherent biases when the instructors assessed CPR performance when using video recordings or checklists by visual assessment [62]. Yet, we extracted the most commonly recognized parameters in CPR quality measurement [63]. Finally, we found that all of studies included had a high risk of bias in the blinding of participants and personnel while performing risk of bias assessment. Because the participants knew the interventions they were performing during evaluation, it might have affected their self-confidence somewhat, influencing CPR performance. This might be another cause for some bias.

## Conclusion

In our systematic review, telephone DA-CPR with simplified or more concrete instructional protocols was shown to improve the quality of bystander CPR. Compression-only CPR and other on-scene interventions also seemed to improve CPR quality. Devices providing real-time feedback and mobile devices containing a CPR app or software were also found to be beneficial to CPR quality. However, using mobile devices for improving CPR quality or for assisting DA-CPR might cause rescuers to spend more time starting CPR. Additional efforts are needed to build up an effective protocol to organize these interventions to improve bystander CPR quality, further improving the clinical outcomes of cardiac arrest victims.

## Supporting information

S1 Table Detailed search strategy. (DOCX)

S2 Table Detailed characteristics of included studies. (DOCX)

S3 Table Detailed outcomes of the included studies. (DOCX)

S4 Table The PRISMA checklist of our study. (DOC)
