# Supplementary material for: Interventions to improve the quality of bystander cardiopulmonary resuscitation: A systematic review
Source: PLoS One. 2019 Feb 13;14(2):e0211792. doi: 10.1371/journal.pone.0211792 (PMC6373936; doi:10.1371/journal.pone.0211792)
Supplement: S1 Table — (DOCX) [file pone.0211792.s001.docx]

**S1 Table. Detailed search strategy**

Search strategy (MEDLINE) 1,222

#1 cardiopulmonary AND (‘resuscitation’/exp OR resuscitation) AND [medline]/lim AND [1966-2018]/py (15,662)

#2 cpr AND [medline]/lim AND [1966-2018]/py (12,814)

#3 heart AND massage [medline]/lim AND [1966-2018]/py (3,624)

#4 cardiac AND massage AND [medline]/lim AND [1966-2018]/py (1,838)

#5 cardiac AND arrest AND [medline]/lim AND [1966-2018]/py (37,010)

#6 heart AND arrest AND [medline]/lim AND [1966-2018]/py (52,999)

#7 cardiopulmonary AND arrest AND [medline]/lim AND [1966-2018]/py (16,623)

#8 sudden AND cardiac AND death AND [medline]/lim AND [1966-2018]/py (26,518)

#9 ‘out of hospital cardiac arrest’ AND [medline]/lim AND [1966-2018]/py (4,981)

#10 ohca AND [medline]/lim AND [1966-2018]/py (1482)

#11 resuscitation AND [medline]/lim AND [1966-2018]/py (78,789)

#12 #1 OR #2 OR #3 OR #4 OR #5 OR #6 OR #7 OR #8 OR #9 OR #10 OR #11 (147,157)

#13 bystander AND [medline]/lim AND [1966-2018]/py (7,804)

#14 layperson AND [medline]/lim AND [1966-2018]/py (387)

#15 public AND [medline]/lim AND [1966-2018]/py (1,252,874)

#16 witness AND [medline]/lim AND [1966-2018]/py (13,699)

#17 ‘non professional’ AND [medline]/lim AND [1966-2018]/py (973)

#18 #13 OR #14 OR #15 OR #16 OR #17 (1,273,339)

#19 quality AND [medline]/lim AND [1966-2018]/py (1,038,117)

#20 compression AND depth AND [medline]/lim AND [1966-2018]/py (1,683)

#21 compression AND rate AND [medline]/lim AND [1966-2018]/py (11,399)

#22 chest AND recoil AND [medline]/lim AND [1966-2018]/py (370)

#23 compression AND fraction AND [medline]/lim AND [1966-2018]/py (1,332)

#24 ‘hands-off time*’ AND [medline]/lim AND [1966-2018]/py (91)

#25 ‘peri-shock pause*’ AND [medline]/lim AND [1966-2018]/py (9)

#26 ‘peri-defibrillation pause*’ AND [medline]/lim AND [1966-2018]/py (0)

#27 ‘post-shock pause*’ AND [medline]/lim AND [1966-2018]/py (12)

#28 ‘post-defibrillation pause*’ AND [medline]/lim AND [1966-2018]/py (2)

#29 ‘pre-shock pause*’ AND [medline]/lim AND [1966-2018]/py (23)

#30 ‘pre-defibrillation pause*’ AND [medline]/lim AND [1966-2018]/py (0)

#31 ‘q-cpr’ AND [medline]/lim AND [1966-2018]/py (18)

#32 ‘qcpr’ AND [medline]/lim AND [1966-2018]/py (13)

#33 #19 OR #20 OR #21 OR #22 OR #23 OR #24 OR #25 OR #26 OR #27 OR #28 OR #29 OR #30 OR #31 OR #32 (1,050,544)

#34 #12 AND #18 AND #33 (1,340)

#35 #12 AND #18 AND #33 AND [animals]/lim (21)

#36 #34 NOT #35 (1,319)

#37 #34 NOT #35 AND ([article]/lim OR [article in press]/lim OR [review]/lim) (1,222)

Search strategy (EMBASE) 1264

#1 cardiopulmonary AND (‘resuscitation’/exp OR resuscitation) AND [embase]/lim AND [1966-2018]/py (21,405)

#2 cpr AND [embase]/lim AND [1966-2018]/py (20,599)

#3 heart AND massage [embase]/lim AND [1966-2018]/py (3,322)

#4 cardiac AND massage AND [embase]/lim AND [1966-2018]/py (1,907)

#5 cardiac AND arrest AND [embase]/lim AND [1966-2018]/py (56,594)

#6 heart AND arrest AND [embase]/lim AND [1966-2018]/py (73,328)

#7 cardiopulmonary AND arrest AND [embase]/lim AND [1966-2018]/py (22,705)

#8 sudden AND cardiac AND death AND [embase]/lim AND [1966-2018]/py (39,795)

#9 ‘out of hospital cardiac arrest’ AND [embase]/lim AND [1966-2018]/py (9,110)

#10 ohca AND [embase]/lim AND [1966-2018]/py (3,822)

#11 resuscitation AND [embase]/lim AND [1966-2018]/py (120,463)

#12 #1 OR #2 OR #3 OR #4 OR #5 OR #6 OR #7 OR #8 OR #9 OR #10 OR #11 (215,316)

#13 bystander AND [embase]/lim AND [1966-2018]/py (10,898)

#14 layperson AND [embase]/lim AND [1966-2018]/py (496)

#15 public AND [embase]/lim AND [1966-2018]/py (1,219,950)

#16 witness AND [embase]/lim AND [1966-2018]/py (9,790)

#17 ‘non professional’ AND [embase]/lim AND [1966-2018]/py (1127)

#18 #13 OR #14 OR #15 OR #16 OR #17 (1,239,642)

#19 quality AND [embase]/lim AND [1966-2018]/py (1,273,382)

#20 compression AND depth AND [embase]/lim AND [1966-2018]/py (2,584)

#21 compression AND rate AND [embase]/lim AND [1966-2018]/py (18,244)

#22 chest AND recoil AND [embase]/lim AND [1966-2018]/py (530)

#23 compression AND fraction AND [embase]/lim AND [1966-2018]/py (2,363)

#24 ‘hands-off time*’ AND [embase]/lim AND [1966-2018]/py (169)

#25 ‘peri-shock pause*’ AND [embase]/lim AND [1966-2018]/py (29)

#26 ‘peri-defibrillation pause*’ AND [embase]/lim AND [1966-2018]/py (0)

#27 ‘post-shock pause*’ AND [embase]/lim AND [1966-2018]/py (35)

#28 ‘post-defibrillation pause*’ AND [embase]/lim AND [1966-2018]/py (3)

#29 ‘pre-shock pause*’ AND [embase]/lim AND [1966-2018]/py (63)

#30 ‘pre-defibrillation pause*’ AND [embase]/lim AND [1966-2018]/py (1)

#31 ‘q-cpr’ AND [embase]/lim AND [1966-2018]/py (62)

#32 ‘qcpr’ AND [embase]/lim AND [1966-2018]/py (62)

#33 #19 OR #20 OR #21 OR #22 OR #23 OR #24 OR #25 OR #26 OR #27 OR #28 OR #29 OR #30 OR #31 OR #32 (1,292,672)

#34 #12 AND #18 AND #33 (2,040)

#35 #12 AND #18 AND #33 AND [animals]/lim (45)

#36 #34 NOT #35 (1,995)

#37 #34 NOT #35 AND ([article]/lim OR [article in press]/lim OR [review]/lim) (1,264)

Search strategy (CINAHL) 794

Limiters: Published Date: 19660101-20181031

Expanders: Apply related words

Search modes: Find all my search items

#1 TX cardiopulmonary resuscitation (8,864)

#2 TX cpr (5,115)

#3 TX heart massage (733)

#4 TX cardiac massage (290)

#5 TX cardiac arrest (7,812)

#6 TX heart arrest (8,701)

#7 TX cardiopulmonary arrest (4,583)

#8 TX sudden cardiac death (5,528)

#9 TX out of hospital cardiac arrest (1,875)

#10 TX ohca (348)

#11 TX resuscitation (20,541)

#12 #1 OR #2 OR #3 OR #4 OR #5 OR #6 OR #7 OR #8 OR #9 OR #10 OR #11 (34,279)

#13 TX bystander (1,596)

#14 TX layperson (451)

#15 TX public (421,957)

#16 TX witness (5,071)

#17 TX non-professional (357)

#18 #13 OR #14 OR #15 OR #16 OR #17 (427,778)

#19 TX quality (318,943)

#20 TX compression depth (345)

#21 TX compression rate (1,828)

#22 TX chest recoil (70)

#23 TX compression fraction (138)

#24 TX hands-off time* (432)

#25 TX peri-shock pause* (2)

#26 TX peri-defibrillation pause* (0)

#27 TX post-shock pause* (1)

#28 TX post-defibrillation pause* (0)

#29 TX pre-shock pause* (0)

#30 TX pre-defibrillation pause* (0)

#31 TX q-cpr (4)

#32 TX qcpr (8)

#33 #19 OR #20 OR #21 OR #22 OR #23 OR #24 OR #25 OR #26 OR #27 OR #28 OR #29 OR #30 OR #31 OR #32 (320,757)

#34 #12 AND #18 AND #33 (794)

Search strategy (Psycinfo) 50

Limiters: Published Date: 19660101-20181031

Expanders: Apply related words

Search modes: Find all my search items

#1 TX cardiopulmonary resuscitation (639)

#2 TX cpr (1,034)

#3 TX heart massage (89)

#4 TX cardiac massage (22)

#5 TX cardiac arrest (939)

#6 TX heart arrest (769)

#7 TX cardiopulmonary arrest (263)

#8 TX sudden cardiac death (910)

#9 TX out of hospital cardiac arrest (96)

#10 TX ohca (18)

#11 TX resuscitation (1,867)

#12 #1 OR #2 OR #3 OR #4 OR #5 OR #6 OR #7 OR #8 OR #9 OR #10 OR #11 (4,225)

#13 TX bystander (2,192)

#14 TX layperson (1,419)

#15 TX public (315,526)

#16 TX witness (11,726)

#17 TX non-professional (649)

#18 #13 OR #14 OR #15 OR #16 OR #17 (329,493)

#19 TX quality (267,149)

#20 TX compression depth (111)

#21 TX compression rate (358)

#22 TX chest recoil (3)

#23 TX compression fraction (18)

#24 TX hands-off time* (39)

#25 TX peri-shock pause* (0)

#26 TX peri-defibrillation pause* (0)

#27 TX post-shock pause* (1)

#28 TX post-defibrillation pause* (0)

#29 TX pre-shock pause* (0)

#30 TX pre-defibrillation pause* (0)

#31 TX q-cpr (0)

#32 TX qcpr (0)

#33 #19 OR #20 OR #21 OR #22 OR #23 OR #24 OR #25 OR #26 OR #27 OR #28 OR #29 OR #30 OR #31 OR #32 (267,602)

#34 #12 AND #18 AND #33 (50)

**Search strategy (Web of science)** 964

Limiters: Published Date: 1966-2018

Source: SCI-EXPANDED

#1 cardiopulmonary resuscitation (19,911)

#2 cpr (11,228)

#3 heart massage (611)

#4 cardiac massage (923)

#5 cardiac arrest (40,309)

#6 heart arrest (15,423)

#7 cardiopulmonary arrest (17,573)

#8 sudden cardiac death (27,663)

#9 out of hospital cardiac arrest (6,564)

#10 ohca (1,673)

#11 resuscitation (52,161)

#12 #1 OR #2 OR #3 OR #4 OR #5 OR #6 OR #7 OR #8 OR #9 OR #10 OR #11 (109,452)

#13 bystander (9,582)

#14 layperson (1,088)

#15 public (338,298)

#16 witness (24,566)

#17 non-professional (1032)

#18 #13 OR #14 OR #15 OR #16 OR #17 (371,885)

#19 quality (1,504,514)

#20 compression depth (6062)

#21 compression rate (39,571)

#22 chest recoil (208)

#23 compression fraction (7,401)

#24 hands-off time* (382)

#25 peri-shock pause* (9)

#26 peri-defibrillation pause* (0)

#27 post-shock pause* (16)

#28 post-defibrillation pause* (3)

#29 pre-shock pause* (34)

#30 pre-defibrillation pause* (0)

#31 q-cpr (19)

#32 qcpr (15)

#33 #19 OR #20 OR #21 OR #22 OR #23 OR #24 OR #25 OR #26 OR #27 OR #28 OR #29 OR #30 OR #31 OR #32 (1,549,196)

#34 #12 AND #18 AND #33 (964)

Search strategy (Cochrane CENTRAL) 230

There are 230 results from 1294100 records

Search statement:

((cardiopulmonary resuscitation) or (cpr) or (heart massage) or (cardiac massage) or (cardiac arrest) or (heart arrest) or (cardiopulmonary arrest) or (sudden cardiac death) or (out of hospital cardiac arrest) or (ohca) or (resuscitation)) and ((bystander) or (layperson) or (public) or (witness) or (non-professional)) and ((quality) or (compression depth) or (compression rate) or (chest recoil) or (compression fraction) or (hands-off time*) or (peri-shock pause*) or (peri-defibrillation pause*) or (post-shock pause*) or (post-defibrillation pause*) or (pre-shock pause*) or (pre-defibrillation pause*) or (q-cpr) or (qcpr))

Limiter:

Publication Year from 1966 to 2018 in Trials
