# Supplementary material for: Interventions to improve the quality of bystander cardiopulmonary resuscitation: A systematic review
Source: PLoS One. 2019 Feb 13;14(2):e0211792. doi: 10.1371/journal.pone.0211792 (PMC6373936; doi:10.1371/journal.pone.0211792)
Supplement: S2 Table — (DOCX) [file pone.0211792.s002.docx]

| **S2 Table. Detailed characteristics of included studies.** | | | | | | | | |
| --- | --- | --- | --- | --- | --- | --- | --- | --- |
| First author  (year published) nation | Subjects | Enrolment | Study design | Study Group | Outcome | Evaluation  methods | Results | funded by a commercial interest |
| Kellermann A et al (1989)  USA [49] | Volunteers (mixed with trained and untrained)  (n=151) | 3 months with unknown date | Non-RCT | (A) volunteers without prior CPR training with telephone instruction (n=65)  (B) previously trained volunteers with telephone instruction (n=43)  (C) previously trained volunteers without telephone instruction (n=43) | Proper actions;  number and volume of ventilations; number, depth and rate of chest compressions | Evaluated by instructors and recording manikins | Group B > Group A > Group C in CPR quality | No |
| Woollard M et al (2003)  UK [25] | Untrained lay persons aged>18 y/o (n=60) | unknown | RCT | (A) compression-only telephone CPR group (n=29)  (B) standard telephone CPR group (n=30) | (1) initial checks and rescue breaths  (2) chest compressions  (3) delay to first compression and number of compression and ventilations delivered  (4) evidence of exhaustion during test  (5) dispatcher compliance to telephone instruction scripts | Observation of the video recording and measurements from a CPR training manikin with software | Ventilation performance poor in Group B.  Compression numbers: Group A > Group B  Less delay to first compression in Group A. No evidence of exhaustion in both groups. | No |
| Williams JG et al (2006)  USA [44] | untrained and healthy non-patient visitors to the ED (n=54) | January 2003-July 2003 | RCT | (A) subjects receiving traditional telephone CPR (n=25)  (B) subjects receiving compressions-only telephone CPR (n=25) | Time to first compression (primary); CPR quality, fatigue and understanding of instruction (secondary) | Stopwatch used to measure time to first compression and recording strips from manikin | Group B had less time paused and time to first compression.  No differences  in perceived  fatigue in both groups. | No |
| Dias JA et al (2007)  USA [34] | Volunteers from a major urban university aged >19 y/o (mixed with trained and untrained)  (n=133) | Unknown | RCT | (A) subjects given standard compression only-CPR (CC-CPR) protocol (n=59)  (B) subjects given simplified CC-CPR protocol (n=58) | Chest compression rate, depth, hand position, full release, overall proportion of compressions without error, time to start of CPR and total hands-off chest time (primary) | Skillreporter^TM^ manikin | Group B had less time to first compression and performed CPR better except hand position. | Partial |
| Brown TB et al (2008)  USA [22] | Volunteers from sites throughout the Birmingham, and the campus of a major urban university, aged>19 y/o (mixed with trained and untrained) (n=215) | Unknown | RCT | (A) subjects without receiving “put the phone down” instructions (n=108)  (B) subjects receiving “put the phone down” instructions (n=107) | Chest compression rate, depth, proportion of compressions without error, with correct hand position, adequate depth, and total release. Time to start of compressions and total hands-off-chest time. (Primary) | Stopwatch and data from the manikin | Group A = Group B except compressions with full release (Group B > Group A) | No |
| Mirza M et al (2008)  USA [37] | Volunteers recruited at public sites throughout central Alabama aged>19 y/o (mixed with trained and untrained) (n=332) | Unknown | RCT | (A) Subjects with the instruction “push down firmly 2 inches” (n=168)  (B) Subjects with the instruction “push as hard as you can” (n=164) | Chest compression rate and depth; proportion of compressions without error, with correct hand position, with adequate depth and with total release. Time to start of CPR and total hands-off-chest time (primary) | Stopwatch and data from the manikin | Group B > Group A in correct compressing depth with the similar proportion of compression rate and full release. | No |
| Nikandish R et al (2008)  Iran [36] | First year public health students (untrained) (n=70) | 2006 | RCT with crossover study | (A) dominant hand group (n=59)  (B) non-dominant hand group (n=59) | Total number of chest compressions, number of correct chest compressions, inadequate compressions, too strong compressions, wrong hand placement, moment of appearance of fatigue and duration of continuous compression | Recording manikin | Group A = Group B in CPR quality. | No |
| Yang CW et al (2008)  Taiwan [28] | Volunteers > 16 y/o without receiving CPR training within 5 years (mixed with trained and untrained) (n=96) | April 2007-July 2007 | RCT | (A) Voice group: only voice CPR instruction via a cell phone (n=53)  (B) Video group: interactive voice and video instruction via a cell phone (n=43) | Opening the airway, making visible chest rise, inflation volume of each rescue breath, time to open the airway, time to first rescue breath and total duration of instruction | Video evaluated by 2 emergency physicians and data from the computer (manikin). | Group B > Group A in quality of rescue breathing. | No |
| Bolle SR et al (2009)  Norway [46] | Students from different high schools (mixed with trained and untrained) (n=180) | Dec. 2006-Jan. 2007 | RCT | (A) Audio group: non-loudspeaker audio-call instruction (n=26)  (B) Video group: loudspeaker video-assisted instruction (n=29)  * The study paired 3 into one group. | Total number of compressions, average depth, rate, time to first compression, total hands-off-chest time, total number of ventilations, average ventilation volume, time to first ventilation. | Video and Data from Skillreporter^TM^ manikin | Group A = Group B | Partial |
| Yang CW et al (2009)  Taiwan [20] | Volunteers > 16 y/o without receiving CPR training within 5 years (mixed with trained and untrained) (n=96) | April 2007-July 2007 | RCT | (A) Voice group: only voice CPR instruction via a cell phone (n=53)  (B) Video group: interactive voice and video instruction via a cell phone (n=43) | Compression rate, depth, proportion of subjects with sufficient rate (>100/min), proportion of chest compressions with appropriate depth (38-51 mm) and correct hand positioning. Hands-off time (pause time > 1.5 sec), time to first chest compression and total duration of CPR instructions. | Video and Data from Skillreporter^TM^ manikin | Group B > Group A in rate and depth of chest compression. Group B had longer time to first chest compression. | No |
| Merchant RM et al (2010)  USA [23] | CPR-trained and untrained veterans and family members/caregivers, aged 18-60 y/o (n=160) | November 2008-Marrch 2009 | RCT | (A) CPR trained, receiving a telephone aid (n=42)  (B) CPR trained, not receiving a telephone aid (n=40)  (C) no CPR training history, receiving a cell telephone aid (n=38)  (D) no CPR training history, not receiving a cell telephone aid (n=40) | Chest compression rate (primary), depth, and hand placement,  Pauses in CPR throughout the session, and time to first compression | Videotape evaluated by two authors and data from Skillreporter^TM^ manikin | Group A+C > Group B+D in all CPR quality, but Group A+C had longer time to first chest compression. | No |
| Neset A et al (2010)  Norway [42] | Lay persons receiving CPR training 5-7 months ago, aged 50-76 y/o (n=64) | Unknown | RCT | (A) CCC with feedback (n=16)  (B) 30:2 with feedback (n=16)  (C) CCC without feedback (n=16)  (D) 30:2 without feedback (n=16) | Compression depth, compression rate, number of compressions per minute, change in compression depth over time, minimum force turning point, ventilation volumes, ventilation rate, number of ventilations, hand-off time, self-reported exhaustion and pain, and attained percentage of age-predicted maximal heart rate. | Data from Skillreporter^TM^ manikin and survey | Group A+C = Group B+D in CPR quality and exhaustion;  Group A+B > Group C+D in CPR quality. | Partial |
| Nishiyama C et al (2010)  Japan [32] | Trained volunteers aged ≧ 18 years recruited from the general public (n=223) | Dec. 2005-July 2006 | RCT | (A) Chest compression-only CPR group (n=106)  (B) Conventional CPR (30:2) group (n=107) | Proportion of chest compressions with appropriate depth among the total chest compressions during every 20-s CPR period (primary); number of chest compressions, time to CPR, and no-flow time (secondary). | Data from Skillreporter^TM^ manikin | Group B > Group A in CPR quality in in 61-80 seconds. Group A had shorter time to first resuscitation and no-flow time. | No |
| Ghuysen A et al (2011)  Belgium [29] | Untrained volunteers recruited in a movie centre; previously trained volunteers recruited in nursing schools (n=110) | January 2009 and April 2009 | RCT | (A) untrained non-guided group (n=30)  (B) untrained guided group (by phone)(n=30)  (C) trained non-guided group (n=25)  (D) trained guided group (by phone) (n=25) | Cardiff evaluation test by two independent raters (primary); a global performance score based on eight binary variables (asking for response, shaking the shoulders, opening the airway, ‘look-listen and feel’ sequence, hand positioning, rate and depth of compressions, thorax relaxation. (secondary) | Cardiff evaluation test and data from Skillreporter^TM^ manikin | Group A < Group B in CPR quality. Group B had longer time to compression. | No |
| Lee JS et al (2011)  Korea [50] | Lay volunteers without previous CPR training with mean age 55-56 y/o (n=78) | May 2010-June 2010 | RCT | (A) Video group: received aid by watching a video on a cellular phone while performing compression-only CPR (n=39)  (B) Audio group:  had the aid of a voice dispatcher while performing compression-only CPR (n=39) | Mean chest compression rate, percentage of the appropriate chest compression rate, mean chest compression depth, percentage of the appropriate chest compression depth, correctness of hand position, time to first chest compression and hands-off time. | Video reviewed by two emergency physicians and data from Skillreporter^TM^ manikin | Group A > Group B in CPR quality. Group A had shorter time to first chest compression and higher percentage of subjects without hand-off event. | No |
| Paal P et al (2012)  Italy [43] | Untrained visitors of a trade fair (n=141) | Unknown | RCT | (A) Assisted BLS group: with the aid of a BLS software program on a mobile phone (n=64)  (B) Non-assisted BLS group: without the aid (n=77) | Overall score evaluated by a score chart (primary); percentage of achievement of BLS steps (secondary) | Skillreporter^TM^ manikin and a score chart. | Group A > Group B in overall score. Group A had longer time to start of compression. | No |
| Rössler B et al (2013)  Austria [51] | Untrained volunteers between 18 and 80 years of age of non-medical profession (n=94) | December 2011-April 2012 | RCT | (A) Non-flowchart group: performed CPR without flowchart support (n=41)  (B) Flowchart group: performed CPR with flowchart support (n=43) | Overall hands-off time in 5-minute CPR (primary); corrected hands-off time from the first set of chest compressions (CC) to the end of the scenario (secondary)  Others: completeness of the BLS assessment, time to CC, total number of CC, CC per cycle, compression depth and compression rate | Evaluated by an independent investigator using a Skillreporter^TM^ manikin | Group B > Group A in completeness of BLS algorithm correctly, overall hands-off time and confidence. | No |
| Birkenes TS et al (2013)  Norway [41] | Volunteers among employees at the local Norwegian Labor and Welfare office and from a youth group from The Norwegian Trekking Association , (mixed with trained and untrained) (n= 38) | April 2010 | RCT | (A) Reference instruction group: based on ERC recommendations (n=19)  (B) Intervention instruction group: using arm and nipple line  (n=18) | Hand position offset | Measured using the laser beam at the upper and lower borders of the compressing hands and photographed | Group B > Group A in hand position. | Full |
| Buléon C et al (2013)  France [45] | Untrained university students (n=164) | 2 months | Randomized crossover controlled trial | (A) Guided group: feedback by the CPRmeter device (n=154)  (B) Blinded group: without feedback by the CPRmeter device (n=154) | Primary endpoint: rate of efficient chest compression (CC) (fulfill all the 3 measures: adequate CC rate (90-120/min), adequate CC depth (≧ 38 mm), and a CC release weight lower than 2500g.  Secondary endpoints: average CC rate, percentage of adequate CC rate (90-120 /min), average CC depth, percentage of adequate CC depth (≧ 38 mm), average CC peak force exerted, average CC release weight exerted, and percentage of adequate CC recoil/release (lower than 2500g). | Data recorded by the CPR meter on a memory microSD card. | Group A > Group B in CPR quality. | Partial |
| Eisenberg Chavez D et al (2013)  USA [52] | Participants recruited from local community centers in Seattle and King County, Washington(mixed with trained and untrained) (n=99) | Unknown | RCT | (A) No dispatch instruction to remove clothing (n=47)  (B) dispatch instruction to remove clothing (n=52) | Time to first chest compression, compression depth, full chest recoil and compression rate | Measured by study coordinator and data from Skillreporter^TM^ manikin. | Group B had longer time to first compression than Group A.  Group A = Group B in CPR quality. | Partial |
| Park SO et al (2013)  Korea [21] | Untrained laypersons aged >18 y/o (n=70) | October 2011-February 2012 | RCT | (A) Metronome group: metronome sounds played to the rescuer through the speaker (n=35)  (B) Control group: without metronome sounds, substituted with repeat verbal encouragement (n=35) | The rate of chest compressions (min) and the numbers of providers with chest compressions at rate of 100-120 compressions/min (primary)  Other outcomes; compression depth (mean value, proportion of compression depth <38 mm and proportion of compression depth >50 mm), compression duty cycle, proportion of incomplete chest release, proportion of abnormal hand positions and total number of chest compression. | Datas from Skillreporter^TM^ manikin | Group A > Group B in compression rate, but Group A < Group B in compression depth | No |
| Birkenes TS et al (2014)  Norway [27] | Trained lay people aged 22-69 y/o. (n=95) | November 2011-January 2012 | RCT | (A) Standard T-CPR group: the rescuer perform CPR most of the time without dispatcher involvement (n=49)  (B) Continuous T-CPR group: New protocol with some added instructions with speakerphone activation, removing obstacles and continuous instruction during CPR (n=46) | chest compression depth, hands-off time, adequate compression rates (90-120/min)  Time intervals of different steps. | Data from computer recorded manikin. Audio and video recordings reviewed by one person for time intervals. | Group B > Group A in compression rate, hands-off time and correct hand position. Group A had shorter time to start of compressions than Group B. | Full |
| Painter I et al (2014)  USA [40] | Seattle and King County residents aged ≧ 40 who spoke English as their primary language (n=86) | July 2010-Aug. 2011 | RCT | (1) Simplified scripts group (n=39)  (2) Conventional scripts group (n=36) | Primary outcomes: time interval from call receipt to the first chest compression and the core metrics of chest compression (chest compression depth, rate, release and interruptions). | All data other than time to first compression were obtained by Skillreporter^TM^ manikin. | Group A ≧ B in CPR quality except correct hand position. Group A had shorter time to first compression than Group B. | No |
| Rodriguez SA et al (2014)  USA [35] | Lay volunteers > 18 y/o with English speaking (mixed with trained and untrained) (n=128) | Unknown | RCT | (1) Push hard group: Given “Push as hard as you can” instructions (n=64)  (2) Two inches group: Given “push down approximately 2 inches” instructions (n=64) | Primary outcome: compression depth (mm)  Secondary outcomes: compression rate, count and percentage of compression with leaning (>2.5 kg) and variables indicating compliance with AHA CPR targets | Data measured by a CPR recording defibrillator | Group A > group B in compression depth and rate but Group A < Group B in chest recoil | Full |
| van Tulder R et al (2014)  Austria [26] | Trained lay volunteers ≧ 18 y/o (n=32) | July 17, 2012 | RCT | (A) Standard instruction group: ”push down firmly 5cm” (n=8)  (B) Repeated standard instruction group: repeating the instruction every 20 s (n=8)  (C) Intensified wording group: “It is very important to push down the chest firmly 5 cm every time” (n=8)  (D) Repeated intensified wording group (n=8) | Primary outcome: relative chest compression depth (absolute compression depth minus the leaning depth in millimeters)  Secondary outcomes: absolute distance (compression depth x compression per minute x 10 min in metres), hands-off time to CPR start (s) and cumulative hands-off times (s/10min) and changes in participants’ vital signs. | Data from simulator manikin | Group A = Group B = Group D > Group C in compression depth. | No |
| Kim YH et al (2015)  Korea [47] | Trained First-year university students (n=93) | May 2013-June 2013 | RCT | (A) Same side group: two rescuers on the same side (n=32)  (B) Opposite side group: two rescuers on the opposite side (n=32) | Hands-off time (primary). Mean compression depth, rate of compressions per min, proportion of abnormal chest recoil, proportion of abnormal hand placement, total number of compression and adequate compression/total compression. | Data from Skillreporter^TM^ manikin | Group A had longer hands-off time. | No |
| Rasmussen SE et al (2017)  Denmark [33] | Volunteer laypersons recruited among employees aged > 18 y/o at several Danish companies (mixed with trained and untrained) (n=128) | September 2014-November 2014 | RCT | (A) Novel protocol group: designed based on previous research and pilot studies (n=61)  (B) Standard protocol (n=64) | Primary endpoint: composite outcome score based on time to first compression, hand position, chest compression depth and rate and hands-off time. Secondary endpoint: individual quality measures. | Data was sampled from the manikin. Video recordings were assessed independently by two ERC certified BLS/AED instructors. | Group A > Group B in CPR quality. | No |
| Sakai T et al (2015)  Japan [19] | Non-medical professional participants aged ≧18 y/o(mixed with trained and untrained) (n=87) | January 2011-March 2011 | RCT | (A) CPR support application group: with the aid of the CPR Support application on a smartphone (n=43)  (B) Control group: without the aid of the CPR Support application on a smartphone (n=41) | The proportion of chest compressions and the total number and appropriateness of the chest compressions. The proportions calling 119 and requesting an AED, the number of chest compressions with an appropriate depth (at least 5 cm), the number of chest compressions with correct hand position, compression depth, time to first resuscitation (the shorter time of initiation of chest compression or ventilation), time to first compression, and time without chest compression. | Data from Skillreporting manikin | Group A > Group B in CPR quality. | No |
| Krikscionaitiene A et al (2016)  Lithuania [30] | Lay-people aged ≧ 50 y/o (mixed with trained and untrained) (n=68) | August 2013-November 2013 | RCT | (A) Control group: Standard hands-only CPR with two-hands chest compression (n=32)  (B) Intervention group: hands-only CPR with Andrew’s manoeuver (four-hands chest compression) (n=34) | Primary: mean compression depth  Secondary: total compression number, compressions with adequate depth, mean compression rate, leaning, heart rate, mean arterial pressure, saturation | Data from Skillreporter^TM^ manikin | Group B > Group A in compression depth. | No |
| Spelten O et al (2016)  Germany [31] | Laypersons between 18 and 65 years were recruited (mixed with trained and untrained) (n=60) | 1 July 2012-30 September 2012 | RCT | (A) U-CPR group: uninstructed CPR (n=20)  (B) DACO-CPR group: dispatcher-assisted compression-only CPR (n=19)  (C) DAF-CPR group: full dispatcher-assisted-CPR including rescue ventilation (n=19) | No-flow-time (NFT), compression depth, total number of compressions, compression frequency, correct hand positioning, correct release after compression, minute respiratory volume | Manikin and software. Hand positioning and head-tilt for ventilation reviewed by two independent investigators via video recordings. | Group B had shorter NFT and better compression frequencies than the other groups. | No |
| Stipulante S et al (2016)  Belgium [39] | untrained volunteers from a high school , aged between 16-25 y/o (n=120) | March 2013 | RCT | (A) t-CPR group: only receiving audio telephone instructions (n=60)  (B) v-CPR group: receiving videoconferencing and perform CPR (n=60) | Primary: results of the Cardiff 3.1 evaluation test and global chest compressions performance score evaluating frequency, depth, and position of the hands.  Second: global performance score on the basis of eight binary variables.  Time-related: timing for evaluation of consciousness, airway management, hands-off time, the time to first chest compression and the total duration of CPR instructions. | Audio-video recordings evaluated by investigators and Skillreporter^TM^ manikin. | Group B > Group A in compression rate and correct hand positioning. Group B had longer time to start of compressions than Group A. | No |
| Torney H et al (2016)  UK  (Experiment 2) [38] | volunteers aged ≧ 15 years (mixed with trained and untrained) (n=140) | Unknown | RCT | (A) CPR rate feedback group (n= 68)  (B) Control group: without CPR rate feedback group (n= 72) | Primary: Mean amplitude of the displacement distance of the potentiometer over the 2-min CPR period  Secondary: Proportion of participants who achieved “good speed” within 45 s, and chest compression fraction | Data from public access defibrillator | Group A > Group B in compression depth, compression rate and compression fraction. | Unknown |
| Hurst V 4^th^ et al (2007)  USA [53] | Trained volunteers, not formally trained in a medical discipline (n=40) | unknown | Cross-over interventional study | (A) BVM group: bag-valve mask with self-inflating bag (n=40)  (B) Model 730 group: a pneumatically powered transport ventilator that is specifically developed for field use by personnel who have a wide range of training and expertise (n=40) | Delivered tidal volumes (ml), delivered airway pressures (cmH2O), delivered airway flow rates (ml/min) and chest compressions. | Data were collected on a laptop computer using devices and software from the research pneumotach system. | Group B > Group A in ventilation quality. | Yes |
| Atkinson PR et al (1999)  UK [17] | Untrained volunteers who were clerical and portering staff at the hospital (n=38) | Unknown | RCT | (A) CPR with no additional instruction (n=9)  (B) CPR with receiving telephone instruction (n=10)  (C) CPR with advice over a video-link (n=10)  (D) CPR with advice by an instructor standing beside them (n=9) | Total number of correct ventilations, total number of correct chest compressions, total number of chest compressions with correct hand position but incorrect depth and time to onset of CPR | The CPR standard was determined by 2 observers and by computerized analysis of manikin recordings. | Group C = Group D > Group B > Group A in CPR quality. | No |
| Liu S et al (2016)  Canada [18] | Participants aged ≧55 y/o (mixed with trained and untrained) (n=63) | July 2010 to August 2010 | Randomized crossover trial | (A) CCC (continuous chest compression) group (n=63)  (B) 30:2 group (n=62) | Number of chest compressions, number of adequate chest compressions(depth ≧ 5 cm), and compression rate (/min) | Recordings from manikin | Group A > Group B in number of total compressions, but quality of group A decreased significantly faster. | No |
| Trenkamp RH et al (2015)  USA [24] | participants recruited by age and sex to approximately match the age distribution of sudden cardiac arrest victims. (n=49) | July to September, 2012 | Observational study | (A) manual group: performing CPR with their hands (n=49)  (B) heel group: performing CPR with their heels (n=49) | Length of time that the subject could perform compliant compressions defined as at a rate of 100 to 120 compressions per minute, 2-inch in depth (primary outcome). | Recordings from the manikin | Group B had longer  duration to perform compliant compressions than Group A. | No |
| Birkenes TS et al (2012)  Norway [48] | Trained lay people aged 50-75 y/o(n=30) | Unknown | Observational study | Intervention: continuous telephone-instructed 30:2 CPR with duration of 10 minutes.  Compare CPR performance within first minute with  those within 10^th^ minute. | Dispatcher communication, compression technique, ventilation technique, depth and rate chest compressions | Video recordings reviewed by researchers and recordings from the manikin | CPR technique and quality within first minute was better than or equal to those within 10th minute. | No |
| White AE et al (2017) Singapore [54] | Teachers from a local  junior college (n=36) | Unknown | Randomized crossover controlled study | 1. Chest compression with CPRcard^TM^ feedback (n=35) 2. Chest compression without CPRcard^TM^ feedback (n=35) | Met both compression rate (100–120 per minute) and depth (at least 5 cm) (primary); Chest compression rate, compression depth (secondary) | CPRcard^TM^ or Resusci Anne’s SimPad® SkillReporter^TM^ | Group A > Group B in met both target compression rate and depth, and proportion of correct compression rate.  Group A = Group B in correct compression depth | No |
| Wutzler A et al (2018) Germany [55] | Participants  without receiving  specific medical training or having any kind  of medical professional working experience. (n=51) | Unknown | Randomized crossover controlled study | (A) Chest compression with audiovisual feedback (n=48)  (B) Chest compression without audiovisual feedback (n=48) | Percentage of optimal chest compression with a rate between 100–120 min–1 and a depth of 50–60 mm. (Primary)  Compression rate, mean compression  Depth, longest interval without any optimal compression, and the percentage of effective compression trials with  a rate of 100–120 min–1and with >80% of compreesion in target depth. (Secondary) | Data from Physio-Control (TrueCPR Report Generator) | Group A > Group B in percentage of chest compression with  correct rate and depth, and effective compression trials:  Group A = Group B in  compression rate and depth;  Group A < Group B in longest interval without optimal chest compression. | No |
| Liu Y et al (2018) China [56] | laypersons without experiencing with basic life support training or real CPR (n=124) | unknown | RCT | 1. hands-only CPR (AHA 2010 guidelines) without feedback (n=42) 2. hands-only CPR (AHA 2015 guidelines) without feedback (n=42) 3. hands-only CPR (AHA 2015 guidelines) with feedback (n=40) | Chest compression depth, rate, complete chest recoil and chest compression fraction | Data from LinkCPR (SunLife, China) | Group C > Group B > Group A in compression depth.  Group A > group B > Group C in compression rate.  Group C > Group A > Group B in Correct ratio of chest compression. | No |
| Eaton G et al (2018) UK [57] | LaypersonsAged ≧ 18 y/o without being a healthcare professional and attending  a CPR training course in the last 6months. | unknown | Randomized crossover study | (A) CPR with PocketCPR (n=118)  (B) CPR without PocketCPR (n=118) | Percentage of correct compression depth (primary), mean compression rate and hand position (secondary) | Data from the manikin software (Laerdal Resuscitation manikin) | Group A > Group B in percentage of correct compression depth  Group A = Group B in mean compression rate and hand position | No |
| Scott G et al (2018) USA [58] | Layperson from four different places in Utah, aged 14 years and  Older (n=156) | Unknown | RCT | 1. CPR under dispatcher’s instruction with the use of the metronome tool (n=85) 2. CPR under dispatcher’s instruction without the use of the metronome tool (n=63) | Compression rate, the compression depth, and percentage of correct compression rate (100-120 /min) and/or  depth (50-60 mm) | Data from simulator manikin | Group A > Group B in correct compression rate    Group A = Group B in correct  compression depth | No |
