# Supplementary material for: Interventions to improve the quality of bystander cardiopulmonary resuscitation: A systematic review
Source: PLoS One. 2019 Feb 13;14(2):e0211792. doi: 10.1371/journal.pone.0211792 (PMC6373936; doi:10.1371/journal.pone.0211792)
Supplement: S3 Table — (DOCX) [file pone.0211792.s003.docx]

| **S3 Table. Detailed outcomes of the included studies.** | | | |  |
| --- | --- | --- | --- | --- |
| First author  (year  published, nation) | Outcome | Outcome evaluated by the reviewers according to a checklist, video recordings, stopwatch | Outcome from the records of manikin | Outcome summary |
| Kellermann A et al (1989)  USA [49] | Proper actions;  number and volume of ventilations; number, depth and rate of chest compressions | Group A vs Group B vs Group C  (1) Depth: A>C, B>C (*p*<0.001)  (2) Compression rate: A>C, B>C (*p*<0.02)  (3) Recoil: Nil  (4) Interruption: Nil  (5) Ventilation:  Generally, A=B>C  (6) Others:  Mean time to first ventilations (min) :A:B:C=2:38 vs 2:29 vs 1:03 (*p*<0.001)  Mean time to first compression (mins) : A:B:C=4.07 vs 3:53 vs 1:15 (*p*<0.001)  Correct hand position: A>C, B>C ( *p*<0.05) | Group A vs Group B vs Group C  (1) Mean depth (mm): A:B:C =18.1 vs 18.4 vs 12.4 (*p*=0.002)  (2) Mean compression rate (n/min): 58 vs 62 vs 77 (*p*<0.001)  (3) Recoil: Nil  (4) Interruption: Nil  (5) Percentage of adequate compressions 26.9 vs 34.4 vs 12.6 (*p*=0.005)  (5) Ventilation:  Mean volume (L): A:B:C =0.70 vs 1.00 vs 0.98 (*p*=0.14)  Percentage of adequate ventilations: 36.0 vs 52.7 vs 58.3 (*p*=0.02)  (7) Others:  Time to first compression (s): Nil  Correct hand position: Nil | Group B > Group A > Group C in CPR quality |
| Woollard M et al (2003)  UK [25] | (1) initial checks and rescue breaths  (2) chest compressions  (3) delay to first compression and number of compression and ventilations delivered  (4) evidence of exhaustion during test  (5) dispatcher compliance to telephone instruction scripts | Group A vs Group B  (1) Depth: Nil  (2) Compression rate: Nil  (3) Recoil: Nil  (4) Interruption: Nil  (5) Ventilation:  Airway opening: 64% vs 50% (*p*=0.293)  Check for airway obstruction: 84% vs 80% (*p*=0.530)  Breathing check: 76% vs 47% (*p*=0.019)  (6) Others:  Median time to first compression (sec) : 184 vs 245 (*p*<0.001)  Median number of chest compressions delivered during test: 461 vs 186 (*p*<0.001) | Group A vs Group B  (1) Depth:  Proportion of subjects compressing at correct depth (40-50 mm): 17% vs 7% (*p*=0.153)  (2) Compression rate:  Proportion of subjects compressing at correct rate (90-110 per min): 21% vs 13% (*p*=0.343)  (3) Recoil: Nil  (4) Interruption: Nil  (5) Deliver correct breath volume: not applicable vs 17%  (5) Others:  Time to first compression (s): Nil  Correct hand position: 14% vs 38% (*p*=0.042)  No evidence of exhaustion in both groups | Ventilation performance poor in Group B.  Compression numbers: Group A > Group B  Less delay to first compression in Group A. No evidence of exhaustion in both groups. |
| Williams JG et al (2006)  USA [44] | Time to first compression (primary); CPR quality, fatigue and understanding of instruction (secondary) | Group A vs Group B  (1) Depth: Nil  (2) Compression rate: Nil  (3) Recoil: Nil  (4) Interruption:  percentage of time paused in 1 st 3 min of CPR: 36 vs 13 (*p*<0.001)  (5) Ventilation: Nil  (6) Others:  Time to first compression (s): 117 vs 72 (*p*<0.001)  Correct hand position: Nil | Group A vs Group B  (1) Depth:  Total number of correct compressions during study period: 10 vs 13 (non-significant)  (2) Compression rate (n/min): 60 vs 58 (non-significant)  (3) Recoil: Nil  (4) Interruption: Nil  (5) Ventilation:  9% of the ventilations in group A had volume of 800-1200 mL  21% of the ventilations in group A had volume of 500-1200 mL  (6) Others:  Time to first compression (s): Nil  Correct hand position: Nil  No evidence of exhaustion in both groups | Group B had less time paused and time to first compression.  No differences  in perceived  fatigue in both groups. |
| Dias JA et al (2007)  USA [34] | Chest compression rate, depth, hand position, full release, overall proportion of compressions without error, time to start of CPR and total hands-off chest time (primary) | Group A vs Group B  (1) Depth: Nil  (2) Compression rate: Nil  (3) Recoil: Nil  (4) Interruption:  (5) Ventilation: Nil  (6) Others:  Time to start of compressions (sec): 78.6 vs 60.9 (*p*<0.001)  Correct hand position: Nil | Group A vs Group B  (1) Depth:  (a)percentage of chest compressions to the correct depth: 3% vs 31% (*p*<0.01)  (b) mean depth of compressions (mm): 29.7 vs 35.6 (*p*<0.01)  (2) Compression rate: 94/min vs 104/min (*p*=0.13)  (3) Recoil:  Proportion with full release: 1 (0.99-1) vs 1 (0.98-1) (*p*=0.09)  (4) Interruption:  total hands-off chest time (sec): 95 vs 69 (*p*<0.001)  (5) Ventilation: Nil  (6) Others:  Time to first compression (s): Nil  percentage of correct hand position: 84% vs 35% (P<0.01) | Group B had less time to first compression and performed CPR better except hand position. |
| Brown TB et al (2008)  USA [22] | Chest compression rate, depth, proportion of compressions without error, with correct hand position, adequate depth, and total release. Time to start of compressions and total hands-off-chest time. (Primary) | Group A vs Group B  (1) Depth: Nil  (2) Compression rate: Nil  (3) Recoil: Nil  (4) Interruption: Nil  (5) Ventilation: Nil  (6) Others:  Time to start of compressions (sec): Nil  Correct hand position: Nil | Group A vs Group B  (1) Depth:  (a) Average compression depth (mm): 33.6 vs 32.8 (*p*=0.60)  (b) Percentage of compressions done to correct depth: 7% vs 12% (*p*=0.67)  (2) Compression rate (n/min): 97.7 vs 98.8 (*p*=0.82)  (3) Recoil:  Percentage of compressions done with full release: 1 (0.99-1) vs 1(1-1) (*p*=0.05)  (4) Interruption:  total hands-off chest time (sec): 71.0 vs 73.0 (*p*=0.48)  (5) Ventilation: Nil  (6) Others:  Time to start of compressions (sec): 65.0 vs 65.0 (*p*=0.96)  Percentage of compressions with correct hand position: 0.87 vs 0.90 (*p*=0.86) | Group A = Group B except compressions with full release (Group B > Group A) |
| Mirza M et al (2008)  USA [37] | Chest compression rate and depth; proportion of compressions without error, with correct hand position, with adequate depth and with total release. Time to start of CPR and total hands-off-chest time (primary) | Group A vs Group B  (1) Depth: Nil  (2) Compression rate: Nil  (3) Recoil: Nil  (4) Interruption: Nil  (5) Ventilation: Nil  (6) Others:  Time to start of compressions (sec): Nil  Correct hand position: Nil | Group A vs Group B  (1) Depth:  (a) Average compression depth (mm): 29.7 vs 36.4 (*p*<0.001)  (b) proportion of compressions done without error: 0% vs 5% (*p*=0.003)  (c) proportion of compressions done to correct depth: 1% vs 32% (*p*<0.001)  (2) Compression rate (n/min):  97.5 vs 99.7 (*p*=0.56)  (3) Recoil:  Proportion of compressions done with full release: 100% vs 100% (*p*=0.14)  (4) Interruption: Nil  (5) Ventilation: Nil  (6) Others:  time to start of compressions (sec): Nil  Correct hand position: Nil | Group B > Group A in correct compressing depth with the similar proportion of compression rate and full release. |
| Nikandish R et al (2008)  Iran [36] | Total number of chest compressions, number of correct chest compressions, inadequate compressions, too strong compressions, wrong hand placement, moment of appearance of fatigue and duration of continuous compression | Group A vs Group B  (1) Depth: Nil  (2) Compression rate: Nil  (3) Recoil: Nil  (4) Interruption: Nil  (5) Ventilation: Nil  (6) Others:  Time to start of compressions (sec): Nil  Correct hand position: Nil | Group A vs Group B  (1) Depth:  Total number of compressions with inadequate depth (mean): 197 vs 196 (*p*=0.9)  (2) Compression rate: nil  (3) Recoil: Nil  (4) Interruption: Nil  (5) Ventilation: Nil  (6) Others:  Time to start of compressions (sec): Nil  Total number of compressions with incorrect hand placement (mean): 45 vs 64 (*p*=0.1) | Group A = Group B in CPR quality. |
| Yang CW et al (2008)  Taiwan [28] | Opening the airway, making visible chest rise, inflation volume of each rescue breath, time to open the airway, time to first rescue breath and total duration of instruction | Group A vs Group B  (1) Depth: Nil  (2) Compression rate: Nil  (3) Recoil: Nil  (4) Interruption: Nil  (5) Ventilation:  (a) opening the airway properly: 58.5% vs 95.3% (*p*<0.01)  (c) visible chest rise: 28.3% vs 65.1% (*p*<0.01)  (d) open airway while giving ventilation: 60.4% vs 88.4% (*p*<0.01)  (6) Others:  Time to start of compressions (sec): Nil  Correct hand position: Nil | Group A vs Group B  (1) Depth: Nil  (2) Compression rate: Nil  (3) Recoil: Nil  (4) Interruption: Nil  (5) Ventilation:  mean volume of ventilation (ml): 322.0 vs 520.5 (*p*<0.01)  (6) Others:  Time to first rescue breath (sec): 102.0 vs 139.0 (*p*<0.01)  Correct hand position: Nil | Group B > Group A in quality of rescue breathing. |
| Bolle SR et al (2009)  Norway [46] | Total number of compressions, average depth, rate, time to first compression, total hands-off-chest time, total number of ventilations, average ventilation volume, time to first ventilation. | Group A vs Group B  (1) Depth: Nil  (2) Compression rate: Nil  (3) Recoil: Nil  (4) Interruption: Nil  (5) Ventilation: Nil  (6) Others:  Time to start of compressions (sec): Nil  Correct hand position: Nil | Group A vs Group B  (1) Depth:  (a) mean depth (mm): 38 vs 37 (*p*=0.83)  (b) done to correct depth (38-51 mm): 31% vs 35% (*p*=0.53)  (2) Compression rate:  mean rate (n/min): 110 vs 114 (*p*=0.75)  (3) Recoil:  done with full release: 100% vs 100% (*p*=0.83)  (4) Interruption:  Total hands-off-chest time (s): 331 vs 303 (*p*=0.05)  (5) Ventilation:  (a) mean ventilation volume (ml): 1356 vs 1163 (*p*=0.74)  (b) Total number of ventilations: 24 vs 28 (*p*=0.50)  (c) Percentage of correct volume (500-800 ml): 6% vs 11% (*p*=0.30)  (6) Others:  time to start of compressions (sec): 102 vs 104 (*p*=0.29)  correct hand position: 50% vs 45% (*p*=0.52) | Group A = Group B |
| Yang CW et al (2009)  Taiwan [20] | Compression rate, depth, proportion of subjects with sufficient rate (>100/min), proportion of chest compressions with appropriate depth (38-51 mm) and correct hand positioning. Hands-off time (pause time > 1.5 sec), time to first chest compression and total duration of CPR instructions. | Group A vs Group B  (1) Depth: Nil  (2) Compression rate: Nil  (3) Recoil: Nil  (4) Interruption: Nil  (5) Ventilation: Nil  (6) Others:  Time to start of compressions (sec): Nil  Correct hand position: Nil | Group A vs Group B  (1) Depth:  (a) median compression depth (mm): 25.0 vs 36.0 (*p*<0.01)  (b) median percentage chest compressions with correct depth: 0% vs 20.0% (*p*<0.01)  (2) Compression rate:  (a) Compression rate (n/min): 63.0 vs 95.5 (*p*<0.01)  (b) chest compressions with sufficient rate: 30.2% vs 46.5% (non-significant)  (3) Recoil: Nil  (4) Interruption:  Hands-off time (s): 0 vs 5.0 (*p*<0.01)  (5) Ventilation: Nil  (6) Others:  time to start of compressions (sec): 116.0 vs 145.0 (*p*<0.01)  Correct hand position:  median percentage of chest compressions with correct hand positioning: 95.6% vs 84.0% (non-significant)  Median of total instruction time (s): 121.0 vs 150.0 (*p*<0.01) | Group B > Group A in rate and depth of chest compression. Group B had longer time to first chest compression. |
| Merchant RM et al (2010)  USA [23]  *The outcomes here we compare are between telephone group [group A + group C] and no telephone group [group B + group D]. | Chest compression rate (primary), depth, and hand placement,  Pauses in CPR throughout the session, and time to first compression | Group A + C vs Group B+D  (1) Depth: Nil  (2) Compression rate: Nil  (3) Recoil: Nil  (4) Interruption:  Total pauses (s): 74 (72-76) vs 89 (80-98)  (5) Ventilation: Nil  (6) Others:  time to start of compressions (sec): 48 (47-49) vs 18 (15-21)  Correct hand position: Nil | Group A + C vs Group B+D  (1) Depth:  (a) Mean depth (mm): 41 (38-44) vs 31 (28-34)  (b) sufficient depth (38-51mm): 49% (40-71) vs 31% (24-40)  (2) Compression rate:  (a) Mean rate (n/min): 100 (97-103) vs 44 (38-50)  (b) sufficient rate (90-120/min): 91% (85-96) vs 3% (0-10)  (3) Recoil: Nil  (4) Interruption: Nil  (5) Ventilation: Nil  (6) Others:  time to start of compressions (sec): Nil  Correct hand position (%): 97% (94-100) vs 75% (68-83) | Group A+C > Group B+D in all CPR quality, but Group A+C had longer time to first chest compression. |
| Neset A et al (2010)  Norway [42] | Compression depth, compression rate, number of compressions per minute, change in compression depth over time, minimum force turning point, ventilation volumes, ventilation rate, number of ventilations, hand-off time, self-reported exhaustion and pain, and attained percentage of age-predicted maximal heart rate. | A. CCC [(A)+(C)] vs 30:2 [(B)+(D)]  (1) Depth: Nil  (2) Compression rate: Nil  (3) Recoil: Nil  (4) Interruption: Nil  (5) Ventilation: Nil  (6) Others:  time to start of compressions (sec): Nil  Correct hand position: Nil  B. Feedback [(A)+(B)] vs no feedback [(C)+(D)]  (1) Depth: Nil  (2) Compression rate: Nil  (3) Recoil: Nil  (4) Interruption: Nil  (5) Ventilation: Nil  (6) Others:  Time to start of compressions (sec): Nil  Correct hand position: Nil | A. CCC [(A)+(C)] vs 30:2 [(B)+(D)]  (1) Median depth (mm): 41vs 42 (*p*=0.14)  (2) Compression rate:  (a) median number of compressions: 948 vs 574 (*p*<0.0005)  (b) median compression rate (n/min): 96 vs 91 (*p*=0.31)  (3) Recoil: Nil  (4) Interruption:  Total hands-off time, median (s): 2 vs 204 (P<0.0005)  (5) Ventilation:  (a) median ventilation rate (/min): N/A vs 4  (b) median ventilation (ml): N/A vs 960  (6) Others:  time to start of compressions (sec): Nil  Correct hand position: Nil  B. Feedback [(A)+(B)] VS No feedback [(C)+(D)]  (1) Median depth (mm): 41 vs 42(*p*=0.27)  (2) Compression rate:  (a) median number of compressions: 817 vs 705 (*p*=0.010)  (b) median compression rate (/min): 101 vs 86 (*p*=0.002)  (3) Recoil: Nil  (4) Interruption:  Total hands-off time (s): 195 vs 210 (*p*=0.65)  (5) Ventilation:  (a) median ventilation rate (/min): 4 vs 2 (*p*=0.026)  (b) median ventilation volume (ml): 902 vs 1395 (*p*=0.009)  (6) Others:  time to start of compressions (sec): Nil  Correct hand position: Nil | Group A+C = Group B+D in CPR quality and exhaustion;  Group A+B > Group C+D in CPR quality. |
| Nishiyama C et al (2010)  Japan [32] | Proportion of chest compressions with appropriate depth among the total chest compressions during every 20-s CPR period (primary); number of chest compressions, time to CPR, and no-flow time (secondary). | (1) Depth: Nil  (2) Compression rate: Nil  (3) Recoil: Nil  (4) Interruption: Nil  (5) Ventilation: Nil  (6) Others:  Time to start of compressions (sec): Nil  Correct hand position: Nil | Group A vs Group B:  (1) Depth:  Proportion of chest compressions with appropriate depth among the total chest compressions during 20-s CPR period  Significant mean difference between 2 groups in 61-80 seconds (58.2% vs 74.3%, *p*=0.003)  (2) Compression rate:  Number of chest compressions: Group A>Group in any stage  (3) Recoil: Nil  (4) Interruption:  mean no-flow time: 32.0 vs 81.0 (*p*<0.001)  (5) Ventilation: Nil  (6) Others:  mean time to first resuscitation (either chest compression or ventilation) (s) : 32.0 vs 35.0 (*p*=0.005)  Correct hand position: Nil | Group B > Group A in CPR quality in in 61-80 seconds. Group A had shorter time to first resuscitation and no-flow time. |
| Ghuysen A et al (2011)  Belgium [29]  *The outcomes we compared here are between group (1) and group (2), excluding group (3) and (4) due to their nursing background. | Cardiff evaluation test by two independent raters (primary); a global performance score based on eight binary variables (asking for response, shaking the shoulders, opening the airway, ‘look-listen and feel’ sequence, hand positioning, rate and depth of compressions, thorax relaxation. (secondary) | Group A vs Group B  (1) Depth: Nil  (2) Compression rate: Nil  (3) Recoil: Nil  (4) Interruption: Nil  (5) Ventilation:  Airway management successful rate: 0% vs 57% (*p*<0.0001)  (6) Others:  mean time to start of compressions (min): 0.27 vs 2.48 (P<0.0001)  Correct hand position: Nil  CPR performance score: 1.3 vs 4.8 (*p*<0.0001) | Group A vs Group B  (1) Mean depth (mm): 32 vs 41.5  (2) Compression rate (n/min): 41.8 vs 59.7 (*p*<0.0001)  (3) Recoil: Nil  (4) Interruption: Nil  (5) Ventilation: Nil  (6) Others:  time to start of compressions (sec): Nil  Correct hand position: 16.7% vs 40% | Group A < Group B in CPR quality. Group B had longer time to compression. |
| Lee JS et al (2011)  Korea [50] | Mean chest compression rate, percentage of the appropriate chest compression rate, mean chest compression depth, percentage of the appropriate chest compression depth, correctness of hand position, time to first chest compression and hands-off time. | Group A vs Group B  (1) Depth: Nil  (2) Compression rate: Nil  (3) Recoil: Nil  (4) Interruption:  (a) Total hands-off time: 11 vs 24 (non-significant)  (b) Percentage of no “hands-off” event after starting compressions: 71.8% vs 46.2% (*p*<0.05)  (5) Ventilation: Nil  (6) Others:  time to start of compressions (sec): 184 vs 211 (*p*<0.01)  Correct hand position: 71.8% vs 43.6% (*p*=0.01) | Group A vs Group B  (1) Depth:  (a) Mean depth (mm): 27.5vs 31.3 (non-significant)  (b) Percentage of adequate compression depth: 20.5% vs 17.9% (non-significant)  (2) Compression rate:  (a) mean compression rate (/min): 99.5 vs 77.4 (*p*<0.01)  (b) Percentage of adequate compression rate: 59% vs 28.2% (*p*<0.01)  (3) Recoil: Nil  (4) Interruption: Nil  (5) Ventilation: Nil  (6) Others:  time to start of compressions (sec): Nil  Correct hand position: Nil | Group A > Group B in CPR quality. Group A had shorter time to first chest compression and higher percentage of subjects without hand-off event. |
| Paal P et al (2012)  Italy [43] | Overall score evaluated by a score chart (primary); percentage of achievement of BLS steps (secondary) | Group A vs Group B  (1) Depth: Nil  (2) Compression rate: Nil  (3) Recoil: Nil  (4) Interruption: Nil  (5) Ventilation: Nil  (6) Others:  time to start of compressions (sec): Nil  Correct hand position: Nil  *Primary endpoint:  Primary endpoint overall score (mean): 19.2 vs 12.9 (*p*<0.001)  *Secondary endpoint: achievement rate of each step  (a) check environment: 64% vs 27% (*p*<0.001)  (b) protect from environmental risks: 70% vs 39% (*p*<0.001)  (c) call for help: 56% vs 27% (*p*<0.001)  (d) open the upper airway: 78% vs 16% (*p*<0.001) | Group A vs Group B  (1) Depth: non-significant  (2) Compression rate:  Correct chest compression rate: 44% vs 14% (*p*<0.001)  (3) Recoil: Nil  (4) Interruption: Nil  (5) Ventilation: non-significant  (6) Others:  mean time to start of compressions (sec): 165.3 vs 87.1 (*p*<0.001)  Correct hand position: non-significant | Group A > Group B in overall score. Group A had longer time to start of compression. |
| Rössler B et al (2013)  Austria [51] | Overall hands-off time in 5-minute CPR (primary); corrected hands-off time from the first set of chest compressions (CC) to the end of the scenario (secondary)  Others: completeness of the BLS assessment, time to CC, total number of CC, CC per cycle, compression depth and compression rate | Group A vs Group B  (1) Depth: Nil  (2) Compression rate: Nil  (3) Recoil: Nil  (4) Interruption: Nil  (5) Ventilation: Nil  (6) Others:  time to start of compressions (sec): Nil  correct hand position: Nil  completeness of BLS algorithm correctly (%): 0% vs 62% (*p*<0.0001)  feeling confidence  5 vs 7 (*p*=0.0009)  fear of harming patients or making a mistake: non-significant | Group A vs Group B  (1) Depth (mean, mm): 41 vs 43 (*p*=0.49)  (2) Compression rate:  (a) total number of compression (mean): 189 vs 200 (*p*=0.55)  (b) rate (/min): 76 vs 78 (*p*=0.75)  (c) mean compressions per cycle: 17 vs 28 (P<0.0001)  (3) Recoil: Nil  (4) Interruption:  (a) mean overall hands-off time (s): 169 vs 147 (*p*=0.024)  (b) corrected hands-off time (s): 146 vs 87 (*p*<0.0001)  (5) Ventilation: Nil  (6) Others:  mean time to start of compressions (sec): 23 vs 60 (*p*<0.0001)  Correct hand position: Nil | Group B > Group A in completeness of BLS algorithm correctly, overall hands-off time and confidence. |
| Birkenes TS et al (2013)  Norway [41] | Hand position offset | Group A vs Group B  (1) Depth: Nil  (2) Compression rate: Nil  (3) Recoil: Nil  (4) Interruption: Nil  (5) Ventilation: Nil  (6) Others:  time to start of compressions (sec): Nil  Correct hand position:  (1) Less caudal hand placement and the difference in mean hand position offset was 47 mm (*p*=0.001)  (2) None in the intervention group placed their hands in the abdominal region vs. 5/18 in the control group (*p*=0.045) | Group A vs Group B  (1) Depth: Nil  (2) Compression rate: Nil  (3) Recoil: Nil  (4) Interruption: Nil  (5) Ventilation: Nil  (6) Others:  time to start of compressions (sec): Nil  Correct hand position: Nil | Group B > Group A in hand position. |
| Buléon C et al (2013)  France [45] | Primary endpoint: rate of efficient chest compression (CC) (fulfill all the 3 measures: adequate CC rate (90-120/min), adequate CC depth (≧ 38 mm), and a CC release weight lower than 2500g.  Secondary endpoints: average CC rate, percentage of adequate CC rate (90-120 /min), average CC depth, percentage of adequate CC depth (≧ 38 mm), average CC peak force exerted, average CC release weight exerted, and percentage of adequate CC recoil/release (lower than 2500g). | Group A vs Group B  (1) Depth: Nil  (2) Compression rate: Nil  (3) Recoil: Nil  (4) Interruption: Nil  (5) Ventilation: Nil  (6) Others:  Time to start of compressions (sec): Nil  Correct hand position: Nil | Group A vs Group B  (1) Depth:  mean depth (mm): 44:36 (*p*<0.001)  percentage of adequate CC depth: 85% vs 43% (*p*<0.001)  (2) Compression rate:  mean rate(/min): 107: 107 (non-significant)  percentage of adequate CC rate: 81% vs 56% (*p*<0.0001)  (3) Recoil:  percentage of adequate CC release: 100%:99% (non-significant)  *Primary endpoint: Rate of efficient compression (%): 71% vs 26% (*p*<0.0001)  (4) Interruption: Nil  (5) Ventilation: Nil  (6) Others:  time to start of compressions (sec): Nil  Correct hand position: Nil | Group A > Group B in CPR quality. |
| Eisenberg Chavez D et al (2013)  USA [52] | Time to first chest compression, compression depth, full chest recoil and compression rate | Group A vs Group B  (1) Depth: Nil  (2) Compression rate: Nil  (3) Recoil: Nil  (4) Interruption: Nil  (5) Ventilation: Nil  (6) Others:  Average time to first chest compressions (sec): 79 vs 109 (*p*<0.001)  Correct hand position: Nil | Group A vs Group B  (1) Mean depth (mm): 40 vs 41 (*p*>0.05)  (2) Mean compression rate (/min): 99 vs 97 (*p*>0.05)  (3) Recoil:  Complete release: 91% vs 95% (*p*>0.05)  (4) Interruption: Nil  (5) Ventilation: Nil  (6) Others:  time to start of compressions (sec): Nil  Correct hand position: Nil | Group B had longer time to first compression than Group A.  Group A = Group B in CPR quality. |
| Park SO et al (2013)  Korea [21] | The rate of chest compressions (min) and the numbers of providers with chest compressions at rate of 100-120 compressions/min (primary)  Other outcomes; compression depth (mean value, proportion of compression depth <38 mm and proportion of compression depth >50 mm), compression duty cycle, proportion of incomplete chest release, proportion of abnormal hand positions and total number of chest compression. | Group A vs Group B  (1) Depth: Nil  (2) Compression rate: Nil  (3) Recoil: Nil  (4) Interruption: Nil  (5) Ventilation: Nil  (6) Others:  Time to start of compressions (sec): Nil  Correct hand position: Nil | Group A vs Group B  (1) Depth:  (a) mean compression depth (mm): 45.9 vs 46.8 (*p*=0.692)  (b) median percentage of compression depth <38 mm: 69.2% vs 15.7% (*p*=0.035)  (c) median percentage of compression depth >51 mm: 13.5 vs 27.2 (*p*=0.308)  (2) Compression rate:  (a) mean compression rate (/min): 111.9 vs 96.7 (*p*=0.018)  (b) providers of correct compression rate: 32% vs 5% (*p*<0.0001)  (c) mean numbers of chest compressions per minute: 109.4 vs 95.9 (P=0.048)  (d) mean total numbers of chest compressions: 439.4 vs 385.1 (*p*=0.040)  (3) Recoil:  median percentage of incomplete chest release: 0.0 vs 0.0 (*p*=0.478)  (4) Interruption: Nil  (5) Ventilation: Nil  (6) Others:  mean time to start of compressions (sec): 35 vs 37 (*p*=0.658)  Correct hand position:  median percentage of abnormal hand positions: 2.7 vs 22.7 (*p*=0.361) | Group A > Group B in compression rate, but Group A < Group B in compression depth |
| Birkenes TS et al (2014)  Norway [27] | chest compression depth, hands-off time, adequate compression rates (90-120/min)  Time intervals of different steps. | Group A vs Group B  (1) Depth: Nil  (2) Compression rate: Nil  (3) Recoil: Nil  (4) Interruption: Nil  (5) Ventilation: Nil  (6) Others:  Median time to first chest compression (sec): 84 vs 144 (*p*<0.001)  Correct hand position: Nil | Group A vs Group B  (1) Depth:  (a) mean absolute depth (mm): 48 vs 47 (*p*=0.83)  (b) Depth ≧ 40 mm (percentage of total compression)(median): 89 vs 80 (*p*=0.83)  (2) Compression rate:  (a) median total compressions: 870 vs 1000 (*p*=0.014)  (b) mean adequate rate (90-120) (percentage of total): 60% vs 87% (*p*<0.001)  (c) mean rate (n/min): 108 vs 106 (*p*=0.41)  (3) Recoil: Nil  (4) Interruption:  median Hands-off time (s): 64 vs 12 (*p*<0.001)  (5) Ventilation: Nil  (6) Others:  time to start of compressions (sec): Nil  Correct hand position (%)(median): 99.7% vs 100% (*p*=0.001) | Group B > Group A in compression rate, hands-off time and correct hand position. Group A had shorter time to start of compressions than Group B. |
| Painter I et al (2014)  USA [40] | Primary outcomes: time interval from call receipt to the first chest compression and the core metrics of chest compression (chest compression depth, rate, release and interruptions). | Group A vs Group B  (1) Depth: Nil  (2) Compression rate: Nil  (3) Recoil: Nil  (4) Interruption: Nil  (5) Ventilation: Nil  (6) Others:  mean time to first compressions (sec): 99 vs 123 (*p*<0.01)  Correct hand position: Nil | Group A vs Group B  (1) Depth:  (a) mean compression depth (mm): 32 vs 25 (*p*<0.05)  (b) percentage of compressions ≧ 38 mm: 33% vs 20% (*p*=0.14)  (2) Compression rate:  (a) mean compression rate(/min): 102 vs 93 (*p*=0.34)  (b) mean percentage of sufficient rate: 24% vs 19% (*p*=0.45)  (c) mean compression fraction: 78 vs 77 (*p*=0.78)  (3) Recoil:  percentage of complete release: 89 vs 92 (*p*=0.62)  (4) Interruption:  (a) mean number of hands-off periods: 5.3 vs 5.4 (*p*=0.95)  (b) mean total hands off time (s): 39 vs 41 (*p*=0.78)  (5) Ventilation: Nil  (6) Others:  time to start of compressions (sec): Nil  mean percentage of correct hand position: 63% vs 86% (*p*<0.01) | Group A ≧ B in CPR quality except correct hand position. Group A had shorter time to first compression than Group B. |
| Rodriguez SA et al (2014)  USA [35] | Primary outcome: compression depth (mm)  Secondary outcomes: compression rate, count and percentage of compression with leaning (>2.5 kg) and variables indicating compliance with AHA CPR targets | Group A vs Group B  (1) Depth: Nil  (2) Compression rate: Nil  (3) Recoil: Nil  (4) Interruption: Nil  (5) Ventilation: Nil  (6) Others:  Time to start of compressions (sec): Nil  Correct hand position: Nil | Group A vs Group B  (1) Depth (mm)(mean):  (a) The first 5 compressions: 34 vs 26(*p*<0.01)  (b) The first 10 compressions: 36 vs 29 (*p*<0.01)  (c) During the entire 2 min episode: 43 vs 36 (*p*<0.01)  (d) percentage of adequate depth (≧ 47 mm): 39% vs 20% (*p*=0.02)  (2) Compression rate (/min):  (a) The first 5 compressions: 76 vs 61 (*p*=0.015)  (b) The first 10 compressions: 81 vs 69 (*p*=0.011)  (c) During the entire 2 min episode: 93 vs 82 (*p*=0.06)  (d) percentage of adequate rate (≧ 100/min): 36% vs 30% (p=0.45)  (3) Recoil:  percentage of subjects who didn’t achieve full chest recoil: 53% vs 75% (*p*=0.01)  (4) Interruption: Nil  (5) Ventilation: Nil  (6) Others:  time to start of compressions (sec): Nil  Correct hand position: Nil | Group A > group B in compression depth and rate but Group A < Group B in chest recoil |
| van Tulder R et al (2014)  Austria [26] | Primary outcome: relative chest compression depth (absolute compression depth minus the leaning depth in millimeters)  Secondary outcomes: absolute distance (compression depth x compression per minute x 10 min in metres), hands-off time to CPR start (s) and cumulative hands-off times (s/10min) and changes in participants’ vital signs. | Group A vs Group B  (1) Depth: Nil  (2) Compression rate: Nil  (3) Recoil: Nil  (4) Interruption: Nil  (5) Ventilation: Nil  (6) Others:  Time to start of compressions (sec): Nil  Correct hand position: Nil | Group A vs Group B  (1) Depth:  (1) mean relative depth (mm): 35 vs 31 vs 25 vs 31  (2) Absolute disance (mm): 43 vs 32 vs 20 vs 22  (2) Compression rate (/min): 93 vs 89 vs 93 vs 101  (3) Recoil:  Leaning depth: 8 vs 7 vs 5 vs 8  (4) Interruption:  mean cumulative hands off (s): 60 vs 134 vs 157 vs 146  (5) Ventilation: Nil  (6) Others:  time to start of compressions (sec): 52 vs 50 vs 47 vs 60  Correct hand position: Nil  Six participants exhausted during 10-mins’ CPR | Group A = Group B = Group D > Group C in compression depth. |
| Kim YH et al (2015)  Korea [47] | Hands-off time (primary). Mean compression depth, rate of compressions per min, proportion of abnormal chest recoil, proportion of abnormal hand placement, total number of compression and adequate compression/total compression. | Group A vs Group B  (1) Depth: Nil  (2) Compression rate: Nil  (3) Recoil: Nil  (4) Interruption: Nil  (5) Ventilation: Nil  (6) Others:  Time to start of compressions (sec): Nil  Correct hand position: Nil | Group A vs Group B  (1) Median depth (mm): 38 vs 37 (*p*=0.616)  (2) Compression rate:  (a) total number of compressions (median): 815 vs 811 (*p*=0.381)  (b) percentage of adequate compression (median): 2.5% vs 1.0% (*p*=0.171)  (3) Recoil:  percentage of incomplete release (median): 16 vs 4 (*p*=0.564)  (4) Interruption:  cumulative hand-off time (s)(mean): 6.6 vs 4.5 (*p*=0.005)  (5) Ventilation: Nil  (6) Others:  time to start of compressions (sec): Nil  Correct hand position:  percentage of incorrect hand position (median): 11% vs 19% (*p*=0.361) | Group A had longer hands-off time. |
| Rasmussen SE et al (2017)  Denmark [33] | Primary endpoint: composite outcome score based on time to first compression, hand position, chest compression depth and rate and hands-off time. Secondary endpoint: individual quality measures. | Group A vs Group B  (1) Depth: Nil  (2) Compression rate: Nil  (3) Recoil: Nil  (4) Interruption: Nil  (5) Ventilation: Nil  (6) Others:  Time to start of compressions (sec): Nil  Correct hand position: Nil  *Overall score (points)(mean):  18.6 vs 17.5 (*p*<0.001) | Group A vs Group B  (1) Mean depth (mm): 58 vs 52 (*p*=0.02)  (2) Mean compression rate (/min): 114 vs 110 (*p*=0.04)  (3) Recoil:  compressions without total recoil (%)(median): 14 vs 8 (*p*=0.06)  (4) Interruption:  hands-off time per min (s)(median): 6 vs 1 (*p*<0.001)  (5) Ventilation: Nil  (6) Others:  time to start of compressions (sec)(median): 65 vs 72 (*p*<0.001)  Correct hand position (%): 61% vs 23% (*p*=0.01) | Group A > Group B in CPR quality. |
| Sakai T et al (2015)  Japan [19] | The proportion of chest compressions and the total number and appropriateness of the chest compressions. The proportions calling 119 and requesting an AED, the number of chest compressions with an appropriate depth (at least 5 cm), the number of chest compressions with correct hand position, compression depth, time to first resuscitation (the shorter time of initiation of chest compression or ventilation), time to first compression, and time without chest compression. | Group A vs Group B  (1) Depth: Nil  (2) Compression rate: Nil  (3) Recoil: Nil  (4) Interruption: Nil  (5) Ventilation: Nil  (6) Others:  Time to start of compressions (sec): Nil  Correct hand position: Nil | Group A vs Group B  (1) Depth:  compression depth (mm)(mean): 35.0 vs 36.7 (*p*=0.492)  number of Chest compressions with appropriate depth (mean): 65.7 vs 41.0 (*p*=0.095)  (2) Compression rate: Nil  (3) Recoil: Nil  (4) Interruption:  time without chest compression (s)(mean): 4.4 vs 63.8 (*p*<0.001)  (5) Ventilation: Nil  (6) Others:  mean time to start of compressions or ventilations (s): 37.1 vs 29.3 (*p*=0.048)  Correct hand position:  number of chest compressions with correct hand position (mean): 109 vs 42.6 (*p*<0.001)  Rate of calling 119: 67.4% vs 46.3% (*p*=0.041)  Rate of requesting AED: 60.5%: 22.0% (*p*<0.001)  Chest compressions performed: 100% vs 75.6% (*p*<0.001)  Number of total chest compressions (mean): 211.6 vs 77.0 (*P*<0.001) | Group A > Group B in CPR quality. |
| Krikscionaitiene A et al (2016)  Lithuania [30] | Primary: mean compression depth  Secondary: total compression number, compressions with adequate depth, mean compression rate, leaning, heart rate, mean arterial pressure, saturation | Group A vs Group B  (1) Depth: Nil  (2) Compression rate: Nil  (3) Recoil: Nil  (4) Interruption: Nil  (5) Ventilation: Nil  (6) Others:  Time to start of compressions (sec): Nil  Correct hand position: Nil | Group A vs Group B  (1) Depth:  mean compression depth (mm): 47.8 vs 54.2 (*p*=0.002)  number of chest compressions with adequate depth (5-6cm) (mean): 188 vs 334 (*p*=0.012)  percentage of chest compression with adequate depth: 46.9% vs 74.8% (*p*=0.003)  (2) Compression rate:  number of total chest compressions (mean): 394 vs 444 (*p*=0.831)  mean chest compression rate (/min)(mean): 97.3 vs 91.0 (*p*=0.352)  (3) Recoil:  percentage of leaning (mean):1.1% vs 0.8% (*p*=0.639)  (4) Interruption:  mean chest compression fraction: 85.6% vs 86% (*p*=0.882)  mean hand-off time (sec): 69.1 vs 67.2 (*p*=0.882)  mean percentage of chest compression duty cycle: 40.8% vs 44 (*p*=0.083)  (5) Ventilation: Nil  (6) Others:  time to start of compressions (sec): Nil  Correct hand position: Nil | Group B > Group A in compression depth. |
| Spelten O et al (2016)  Germany [31] | No-flow-time (NFT), compression depth, total number of compressions, compression frequency, correct hand positioning, correct release after compression, minute respiratory volume | Group A vs Group B vs Group C  (1) Depth: Nil  (2) Compression rate: Nil  (3) Recoil: Nil  (4) Interruption: Nil  (5) Ventilation: Nil  (6) Others:  time to start of compressions (sec): Nil  Correct hand position: Nil | Group A vs Group B vs Group C  (1) Depth (mm)(mean): 40.6 vs 41.0 vs 38.8 (*p*>0.05)  (2) Mean compression rate (1/min): 35.6 vs 65.5 vs 44.5 (*p*=0.001)  (3) Recoil:  percentage of compressions without correct release(mean): 13.2% vs 16.9% vs 6.5% (*p*>0.05)  (4) Interruption:  overall no-flow-time(sec)(mean): 273.4 vs 99.8 vs 240.1 (p<0.001)  (5) Ventilation:  total number of ventilation attempts (mean): 37.44 vs 2.0 vs 23.26 (group A: C: *p*=0.006)  (6) Others:  time to start of compressions (sec)(mean): 25.1 vs 55.2 vs 101.2 (group B:C:*p*<0.001)  percentage of compressions with wrong hand position (mean): 15.6% vs10.5% vs 16.0% (*p*>0.05)  Number of total compressions (mean): 293.53 vs 512.11 vs 356.53 (*p*=0.001) | Group B had shorter NFT and better compression frequencies than the other groups. |
| Stipulante S et al (2016)  Belgium [39] | Primary: results of the Cardiff 3.1 evaluation test and global chest compressions performance score evaluating frequency, depth, and position of the hands.  Second: global performance score on the basis of eight binary variables.  Time-related: timing for evaluation of consciousness, airway management, hands-off time, the time to first chest compression and the total duration of CPR instructions. | Group A vs Group B  (1) Depth: Nil  (2) Compression rate: Nil  (3) Recoil: Nil  (4) Interruption: Nil  (5) Ventilation:  (a) Open the airway successfully: 68% vs 98.3% (*p*<0.0001)  (b) Breathing check: 82% vs 98.3% (*p*=0.003)  (6) Others:  Time to start of compressions (sec): Nil  Correct hand position: Nil  Time for responsiveness check (s)(median): 34.5 vs 39 (*p*=0.0043)  Time for airway opening (s)(median): 72 vs 66.5 (*p*=0.18)  Time for breathing check (s): 85 vs 93.5 (*p*=0.08) | Group A vs Group B  (1) Depth:  compression depth (mm)(mean): 47.1 vs 48.38 (*p*=0.64)  percentage of compression with appropriate depth: 40.3% vs 43.3% (*p*=0.85)  (2) Compression rate:  mean Compression rate (/min): 85.6 vs 110.4 (*p*<0.0001)  Total number of chest compressions (median): 301 vs 421 (*p*<0.0001)  percentage of CC with appropriate rate: 37.9% vs 80% (*p*<0.0001)  (3) Recoil: Nil  (4) Interruption:  hands-off time (s)(median): 7 vs 0 (*p*<0.0001)  (5) Ventilation: Nil  (6) Others:  time to start of compressions (sec)(median): 122.5 vs 146 (*p*<0.0001)  Correct hand position:  percentage of compressions with correct hand positioning: 68% vs 91.7% (*p*=0.0017) | Group B > Group A in compression rate and correct hand positioning. Group B had longer time to start of compressions than Group A. |
| Torney H et al (2016)  UK  (Experiment 2) [38] | Primary: Mean amplitude of the displacement distance of the potentiometer over the 2-min CPR period  Secondary: Proportion of participants who achieved “good speed” within 45 s, and chest compression fraction | Group A vs Group B  (1) Depth: Nil  (2) Compression rate: Nil  (3) Recoil: Nil  (4) Interruption: Nil  (5) Ventilation: Nil  (6) Others:  time to start of compressions (sec): Nil  Correct hand position: Nil | Group A vs Group B  (1) Depth:  mean depth (mm): 24.61 vs 20.08 (p = 0.001)  (2) Compression rate:  percentage of participants achieving good CPR compression speed within 45s: 95.6% vs 62.5% (*p*<0.0001)  (3) Recoil: Nil  (4) Interruption:  mean percentage chest compression fraction: 91.6% vs 88.7% (non-significant)  (5) Ventilation: Nil  (6) Others:  time to start of compressions (sec): Nil  Correct hand position: Nil | Group A > Group B in compression depth, compression rate and compression fraction. |
| Hurst V 4th et al (2007)  USA [53] | Delivered tidal volumes (ml), delivered airway pressures (cmH2O), delivered airway flow rates (ml/min) and chest compressions. | Group A vs Group B  (1) Depth: Nil  (2) Compression rate: Nil  (3) Recoil: Nil  (4) Interruption: Nil  (5) Ventilation: Nil  (6) Others:  time to start of compressions (sec): Nil  Correct hand position: Nil | Group A vs Group B  (1) Depth: Nil  (2) Compression rate:  average number of compressions (4 min cycle)(mean): 281.85 vs 230.75 (*p*<0.05)  (3) Recoil: Nil  (4) Interruption: Nil  (5) Ventilation:  average number of breaths (4 min cycle)(mean): 38.1 vs 32.0 (*p*<0.05)  average delivered tidal volume per breath (ml)(mean): 803.03 vs 672.08 (*p*<0.05)  average delivered airway flow rate per breath (ml/min-breath)(mean): 161.01 vs 21.31 (*p*<0.05)  average delivered airway pressure per breath (cmH2O)(mean): 14.43 vs 7.54 (*p*<0.05)  (6) Others:  time to start of compressions (sec): Nil  Correct hand position: Nil | Group B > Group A in ventilation quality. |
| Atkinson PR et al (1999)  UK [17] | Total number of correct ventilations, total number of correct chest compressions, total number of chest compressions with correct hand position but incorrect depth and time to onset of CPR | Group A vs Group B vs Group C vs Group D  (1) Depth: Nil  (2) Compression rate: Nil  (3) Recoil: Nil  (4) Interruption: Nil  (5) Ventilation:  Total number of correct ventilations (median): 0 vs 8.5 vs 2.5 vs 2 (compared with group A, the other groups were all significantly difference.)  (6) Others:  Time to start of compressions (sec): Nil  Correct hand position: Nil | Group A vs Group B vs Group C vs Group D  (1) Depth:  (2) Compression rate:  Total number of correct chest compressions (median): 0 vs 0.5 vs 7.5 vs 10 (Group A: Group B, *p*=0.11; Group A: Group C, *p*=0.021; Group A: Group D, *p*=0.046)  (3) Recoil: Nil  (4) Interruption: Nil  (5) Ventilation: Nil  (6) Others:  time to onset of CPR (sec)(median): 7 vs 30 vs 35 vs 34 (Compared with group A, the other groups were all significantly difference.)  Correct hand position:  Total number of compressions with correct hand position but incorrect depth (median): 2 vs 16.5 vs 35 vs 43(Group A: Group B, *p*=0.23; Group A: Group C, *p*=0.023; Group A: Group D, *p*=0.002) | Group C = Group D > Group B > Group A in CPR quality. |
| Liu S et al (2016)  Canada [18] | Number of chest compressions, number of adequate chest compressions(depth ≧ 5 cm), and compression rate (/min) | Group A vs Group B  (1) Depth: Nil  (2) Compression rate: Nil  (3) Recoil: Nil  (4) Interruption: Nil  (5) Ventilation: Nil  (6) Others:  Time to start of compressions (sec): Nil  Correct hand position: Nil | Group A vs Group B  (1) Depth:  number of adequate chest compressions (≧ 5cm in depth)(mean): 381.5 vs 324.9 (*p*=0.0001)  The number of adequate chest compressions decreased over time in CCC group (*p*<0.0001) but not 30:2 group (p=0.75)  (2) Compression rate:  number of chest compressions (mean): 480 vs 376.3 (*p*<0.0001)  compression rate (/min)(mean): 99.7 vs 101.8 (*p*=0.0002)  (3) Recoil: Nil  (4) Interruption: Nil  (5) Ventilation: Nil  (6) Others:  time to start of compressions (sec): Nil  Correct hand position: Nil | Group A > Group B in number of total compressions, but quality of group A decreased significantly faster. |
| Trenkamp RH et al (2015)  USA [24] | Length of time that the subject could perform compliant compressions defined as at a rate of 100 to 120 compressions per minute, 2-inch in depth (primary outcome). | Group A vs Group B  (1) Depth: Nil  (2) Compression rate: Nil  (3) Recoil: Nil  (4) Interruption: Nil  (5) Ventilation: Nil  (6) Others:  Time to start of compressions (sec): Nil  Correct hand position: Nil | Group A vs Group B  (1) Depth:  (2) Compression rate:  (a) percentage of performing compliant compressions for 10 minutes: 16% vs 65%  (b) percentage of subjects performing compressions without adequate depth: 24% vs 2%  (3) Recoil: Nil  (4) Interruption: Nil  (5) Ventilation: Nil  (6) Others:  time to start of compressions (sec): Nil  Correct hand position: Nil  Length of time to perform compliant compressions (sec)(mean): 2.9 vs 7.9 (*p*<0.001) | Group B had longer  duration to perform compliant compressions than Group A. |
| Birkenes TS et al (2012)  Norway [48] | Dispatcher communication, compression technique, ventilation technique, depth and rate chest compressions | *1^st^ min VS 10^th^ min  (1) Depth: Nil  (2) Compression rate: Nil  (3) Recoil: Nil  (4) Interruption: Nil  (5) Ventilation:  (a) Chin lift: 17/29 vs 18/29 (*p*=1.0)  (b) Head lift: 14/29 vs 20/29 (*p*=0.15)  (c) Nose pinch: 19/29 vs 22/29 (*p*=0.25)  (6) Others:  Time to start of compressions (sec): Nil  Hand placement on nipple line: 17/29 vs 24/29 (*p*=0.065)  Communication (participants communicating with dispatcher and performing CPR simultaneously): 29/30 vs 29/30 (non-significant)  Correct rescuer position for chest compressions: 13/30 vs 21/30 (*p*=0.008) | *1^st^ min VS 10^th^ min  (1) Mean compression depth (mm): 43 vs 42 (non-significant)  (2) Mean compression rate (/min): 84 vs 101 (*p*<0.001)  (3) Recoil: Nil  (4) Interruption:  Mean time between compression series (s): 20.5 vs 12.1 (*p*<0.001)  (5) Ventilation:  percentage of participants achieving successful ventilations: 13/30 vs 23/30 (*p*=0.006)  (6) Others:  time to start of compressions (sec): Nil  Correct hand position: Nil | CPR technique and quality within first minute was better than or equal to those within 10th minute. |
| White AE et al (2017) Singapore [54] | Met both compression rate (100–120 per minute) and depth (at least 5 cm) (primary); Chest compression rate, compression depth (secondary) | Group A vs Group B   1. Depth: Nil 2. Compression rate: Nil 3. Recoil: Nil 4. Interruption: Nil 5. Ventilation: : Nil 6. Others: : Nil   Time to start of compressions (sec) : Nil  Correct hand position: Nil | Group A vs Group B   1. Depth (cm) (median):  5.0 : 5.0 (*p*=0.319) 2. Compression rate (/min) (median):  117: 122 (*p*=0.001) 3. Recoil: Nil 4. Interruption: Nil 5. Ventilation: : Nil 6. Others: : Adequate compression rate (median) 83% : 47% (*p*< 0.001) Adequate depth (median) 52% : 48% (*p*=0.957) Met compression rate of 100–120/min & depth ≥ 5 cm(n, %) 9 (36%) : 1 (4%) (*p*=0.022)   Time to start of compressions (sec) : Nil  Correct hand position: Nil | Group A > Group B in met both target compression rate and depth, and proportion of correct compression rate.  Group A = Group B in correct compression depth |
| Wutzler A (2018) Germany [55] | Percentage of optimal chest compression with a rate between 100–120 min–1 and a depth of 50–60 mm. (Primary)  Compression rate, mean compression  Depth, longest interval without any optimal compression, and the percentage of effective compression trials with  a rate of 100–120 min–1and with >80% of compreesion in target depth. (Secondary) | Group A vs Group B   1. Depth: Nil 2. Compression rate: Nil 3. Recoil: Nil 4. Interruption: Nil 5. Ventilation: : Nil 6. Others: : Nil   Time to start of compressions (sec) : Nil  Correct hand position: Nil | Group A vs Group B   1. Depth (mm)(mean): 54: 55.6 (*p*=0.789) 2. Compression rate(/min)(mean): 98.4: 95.7 (*p*=0.937) 3. Recoil: Nil 4. Interruption: Nil 5. Ventilation: : Nil 6. Others: :  Percentage of optimal chest compression(%)(mean) 58.9:14.6 (*p* < 0.0001) Longest interval without optimal chest compression (sec) (mean)  27.5 : 76.5 (*p* < 0.0001) Effective chest compression trials (%)  45.8 : 0 (*p* < 0.0001)   Time to start of compressions (sec) : Nil  Correct hand position: Nil | Group A > Group B in percentage of chest compression with  correct rate and depth, and effective compression trials:  Group A = Group B in  compression rate and depth;  Group A < Group B in longest interval without optimal chest compression. |
| Liu Y et al (2018) China [56] | Chest compression depth, rate, complete chest recoil and chest compression fraction | Group A vs Group B vs Group C  (1) Depth: Nil  (2) Compression rate: Nil  (3) Recoil: Nil  (4) Interruption: Nil  (5) Ventilation: : Nil  (6) Others: : Nil  Time to start of compressions (sec) : Nil  Correct hand position: Nil | Group A vs Group B vs Group C   1. Compression depth (mm)(mean) 1 min: 49 : 51 : 56 (*p* <0.05) 2 min: 44 : 49 : 56 (*p* <0.05) 2. Compression rate (/min)(mean) 1 min: 118: 112 : 104 (*p* <0.05) 2 min: 115: 109 : 104 (*p* <0.05) 3. Recoil:nil 4. Interruption: Nil 5. Ventilation: Nil 6. Others: percentage of correct chest compression depth(%)(mean) 1 min: 63.1: 37.6: 89.1 (*p* <0.05) 2 min: 64.2: 35.8: 88.4 (*p* <0.05) percentage of correct chest compression rate (%)(mean) 1 min 83.5: 61.9: 86.5 (*p* <0.05) 2 min: 76.4: 58.9: 85.9 (*p* <0.05) percentage of correct chest compression (%)(mean) 1 min: 54.9: 29.6: 87.8 (*p* <0.05) 2 min: 53.6: 25.6: 87.1 (*p* <0.05)   Time to start of compressions (sec)  Correct hand position: Nil | Group C > Group B > Group A in compression depth.  Group A > group B > Group C in compression rate.  Group C > Group A > Group B in Correct ratio of chest compression. |
| Eaton G et al (2018) UK [57] | Percentage of correct compression depth (primary), mean compression rate and hand position (secondary) | Group A vs Group B  (1) Depth: Nil  (2) Compression rate: Nil  (3) Recoil: Nil  (4) Interruption: Nil  (5) Ventilation: : Nil  (6) Others: : Nil  Time to start of compressions (sec) : Nil  Correct hand position: Nil | Group A vs Group B   1. Depth: Percentage of mean correct compression depth (5-6mm)(mean)  44.28%: 40.57% (*p* = 0.001) 2. Compression rate(/min)(mean) 106.87 : 105.37 (*p* = 0.858) 3. Recoil: Nil 4. Interruption: Nil 5. Ventilation: : Nil 6. Others:  Total compression(/2min)(mean): 205.19 : 163.25 (*p* < 0.001)   Time to start of compressions (sec) : Nil  Correct hand position: Nil  Low-hand position (%) 23.44 : 44.97 (*p* = 0.970)  High-hand position (%) 15.84 : 17.32 (*p* = 0.351)  Right-hand position (%) 5.56: 2.7 (*p* = 0.194)  Left-hand position(%) 13.82 : 14.56 (*p* = 0.788) | Group A > Group B in percentage of correct compression depth  Group A = Group B in mean compression rate and hand position |
| Scott G et al (2018) USA [58] | Compression rate, the compression depth, and percentage of correct compression rate (100-120 /min) and/or  depth (50-60 mm) | Group A vs Group B   1. Depth: Nil 2. Compression rate: Nil 3. Recoil: Nil 4. Interruption: Nil 5. Ventilation: : Nil 6. Others: : Nil   Time to start of compressions (sec) : Nil  Correct hand position: Nil | Group A vs Group B   1. Correct compression depth (5-6mm), n (%) 4 (4.7%) : 2 (3.2%) 2. Correct compression rate: achieved target rate (100-120/min), n (%) 39 (45.9%) : 14 (22.2%) (*p* = 0.003) 3. Recoil: Nil 4. Interruption: Nil 5. Ventilation: : Nil 6. Others: : Nil   Time to start of compressions (sec) : Nil  Correct hand position: Nil | Group A > Group B in correct compression rate    Group A = Group B in correct  compression depth |
